# Alpha‐Ketoisocaproate Attenuates Muscle Atrophy in Cancer Cachexia Models

**DOI:** 10.1002/jcsm.70044

**Published:** 2025-08-14

**Authors:** Pooreum Lim, Sang Woo Woo, Jihye Han, Young Lim Lee, Jin Ju Lim, Yeong Hoon Kang, Ji Wook Moon, Jeong Min Nam, Jeong Hyeon Kim, Donghun Kim, Jae Ho Shim, Hyeon Soo Kim

**Affiliations:** ^1^ Department of Anatomy Korea University College of Medicine Seoul Republic of Korea

**Keywords:** Akt, alpha‐ketoisocaproate, cancer cachexia, FoxO3a, myostatin, protein turnover

## Abstract

**Background:**

Cancer‐associated cachexia (CAC) is a multifactorial syndrome characterised by progressive loss of muscle mass with limited Food and Drug Administration treatments. Although emerging evidence suggests that l‐leucine and β‐hydroxy‐β‐methyl butyrate (HMB) have potential for treating CAC, the role of α‐ketoisocaproate (KIC), a metabolite of l‐leucine, remains unclear. Therefore, this study explored the use of KIC as a therapeutic agent for CAC‐induced muscle atrophy by targeting myostatin.

**Methods:**

We evaluated the effect of KIC on muscle atrophy using BALB/c mice and C2C12 myotubes as models of C26‐ and 4T1‐induced CAC. Male and female mice were injected with C26 and 4T1 cells, respectively. Grip strength was measured weekly, and mice were sacrificed 4 weeks post‐injection for muscle collection. C2C12 myotubes were treated with conditioned media (CM) derived from C26 or 4T1 cells.

**Results:**

KIC suppressed mRNA expression of myostatin, a key regulator of muscle atrophy, more effectively than did l‐leucine (−26.37 ± 4.11%, *p* < 0.01). KIC enhanced protein turnover in C2C12 myotubes and maintained 50% cell viability at high concentrations (KIC: 4.68 mM, HMB: 3.11 mM). Following CM treatment, KIC suppressed MuRF1 and MAFbx expression in a myostatin‐dependent manner, thereby reducing their polyubiquitination. KIC restored Akt‐FoxO3a phosphorylation, leading to improved myotube diameter (+63.8 ± 25.71%, *p* < 0.05) and fusion index (+51.9 ± 22.6%, *p* < 0.05). Immunofluorescence and nuclear fractionation revealed that KIC reduced FoxO3a nuclear accumulation. CM reduced p‐Akt–FoxO3a interaction, which was rescued by KIC. In vivo, KIC administration increased body weight (11.11 ± 8.53%), grip strength (24.76 ± 10.58%), and skeletal muscle mass (*p* < 0.001) in C26 tumour‐bearing mice. Protein expression of myostatin in the tibialis anterior (TA) muscle (−23.57 ± 12.22%, *p* < 0.05) and serum (−52.11 ± 3.56%, *p* < 0.001) was lower in KIC‐treated mice (*n* = 12) compared with that in the controls. KIC increased the mean fibre cross‐sectional area in TA (24.51 ± 14.14%, *p* < 0.01). In 4T1 tumour‐bearing mice, KIC improved body weight (13.10 ± 10.76%) and grip strength (7.42 ± 4.33%) (*p* < 0.001, *n* = 10). Serum myostatin levels (−57.43 ± 9.46%, *p* < 0.001) and skeletal muscle weight were reduced in KIC‐treated mice (*n* = 10).

**Conclusion:**

Our findings demonstrate that KIC improves muscle function in CAC‐induced muscle atrophy by regulating myostatin expression in skeletal muscle via the Akt–FoxO3a pathway. Thus, KIC has been proposed as a potential therapeutic agent against CAC.

**Abbreviations**
KIC: α‐ketoisocaproate
BCAA: branched‐chain amino acid
CAC: cancer cachexia
CM: Conditioned medium
CSA: cross‐sectional area
DM: differentiation medium
EDL: extensor digitorum longus
FoxO3a: Forkhead box O3
GCM: gastrocnemius
HMB: β‐hydroxy‐β‐methylbutyrate
MAFbx: muscle atrophy F‐box
MCT: monocarboxylic acid transporter
MuRF1: muscle RING‐finger protein 1
QA: quadriceps
SOL: soleus
TA: tibialis anterior
UPS: ubiquitin–proteasome system

## 1 Background

Cancer‐associated cachexia (CAC), affecting 50%–80% of patients with advanced cancer, significantly worsens quality of life and is directly responsible for up to 40% of cancer‐related deaths [1]. CAC is a multifactorial syndrome characterised by progressive weight and skeletal muscle loss; it is more prevalent in patients with digestive cancers such as pancreatic and colon cancer [2]. Despite clinical studies on CAC, treatments approved by the Food and Drug Administration are limited by side effects and low efficacy [3]. Therefore, interventions for CAC should focus on minimising side effects, with an emphasis on safety and efficacy [4].

CAC is associated with a systemic inflammatory state that upregulates protein degradation pathways. A key pathway for skeletal muscle protein degradation is the ubiquitin‐proteasome system (UPS), including atrogenes such as MuRF1 and MAFbx, which act as E3 ubiquitin ligases [5]. Among its regulators, myostatin plays a central role by binding with ActRIIB to stimulate atrogene expression and induce muscle loss [6]. Myostatin overexpression has been observed in patients with chronic diseases, including cancer, and its dysregulation disrupts protein turnover in skeletal muscle, exacerbating CAC‐related muscle atrophy [7, 8]. Therefore, myostatin‐inhibitory interventions have been proposed as a potential therapeutic strategy to ameliorate CAC in patients with cancer [5, 9].

Recent studies emphasise that nutritional interventions such as vitamin D and branched‐chain amino acid (BCAA), particularly l‐leucine, can improve protein balance in CAC [10, 11]. l‐leucine activates Akt/mTOR signalling and reduces E3 ubiquitin ligase expression, but excessive intake can increase plasma ammonia levels and promote cancer growth [10, 12]. Therefore, recent studies have focused on metabolites to improve the side effects of l‐leucine and to identify their role in CAC [13]. β‐Hydroxy‐β‐methylbutyrate (HMB), a metabolite of l‐leucine, enhances muscle protein synthesis through mTOR activation and improves muscle mass and function in patients with cancer [14, 15]. However, the effect of α‐ketoisocaproate (KIC), another metabolite of l‐leucine, on CAC remains unclear. In this study, we report that KIC attenuates CAC‐induced muscle atrophy by inducing FoxO3a nuclear export and that this phenomenon is regulated in an Akt‐dependent manner. Our results reveal an Akt‐FoxO3a‐myostatin axis that underlies muscle atrophy in CAC. Therefore, we suggest that KIC, through the Akt‐FoxO3a pathway, is a potential therapeutic agent for mitigating muscle atrophy in CAC.

## 2 Methods

### 2.1 Reagents

KIC (#68255, TCI, Japan), l‐leucine (#PHR1105), HMB (#H0701), cycloheximide (CHX) (protein synthesis inhibitor, #239763), and LY294002 (LY) (#L9908) (Sigma, USA) were used. MG132 (proteasome inhibitor, #HY‐13259) and AR‐C155858 (ARC) (#HY‐13248) were purchased from MedChemExpress (USA), while puromycin (#631305) was purchased from Takara Bio (USA). Antibodies against anti‐myostatin (#GTX32624, 1:1000; GENTEX, USA), anti‐puromycin (#ARC58626, 1:1000; Abclonal, USA), anti‐MuRF1 (#ab172479, 1:1000), anti‐MAFbx (#ab168372, 1:1000), and anti‐14‐3‐3 (#ab6081, 1:1000) (Abcam, UK) were used. Anti‐ubiquitin (#3936, 1:1000), anti‐p‐Akt^(Ser473)^ (#4060, 1:1000), anti‐Akt (#9272, 1:1000), and anti‐p‐FoxO3a^(Ser253)^ (#9466, 1:1000) antibodies were purchased from CST. Anti‐FoxO3a (#12829, CST) antibody was used at 1:1000 (immunoblotting, IB), 1:200 (immunocytochemistry, ICC), and 1:100 (immunoprecipitation, IP). Anti‐myosin heavy chain (MyHC) (#MAB4470SP, 1:200, ICC) and recombinant myostatin (#788‐G8) were purchased from R&D Systems (USA). Anti‐lamin B (#sc‐6217, 1:1000) and rabbit anti‐IgG (#sc‐2027, 1:100) antibodies were purchased from SantaCruz (USA). Anti‐β‐actin (#E12–041‐4, 1:5000, Enogene, USA), HRP‐conjugated anti‐mouse (#ADI‐SAB‐100‐J, 1:5000), and HRP‐conjugated anti‐rabbit (#ADI‐SAB‐300‐J, 1:3000) (ENZO, USA) were also used.

### 2.2 Cell Culture

Mouse C2C12 (ATCC) and human skeletal muscle cells (HSkM, ThermoFisher) were cultured in DMEM (WELGENE) with 10% FBS (Gibco) and 1% penicillin–streptomycin at 37°C, 5% CO₂. When the C2C12 cells reached 60%–70% confluency, they were subcultured and used for no more than 10 passages. To induce myogenic differentiation, the growth medium was replaced with differentiation medium (DM) containing DMEM supplemented with 2% horse serum (Gibco) when the C2C12 myoblasts reached 90%–100% confluency. Mouse C26 (colon carcinoma, BioLabs) and 4T1 (mammary carcinoma, ATCC) cells were cultured in RPMI1640 (WELGENE) supplemented with 10% FBS and 1% penicillin–streptomycin. Conditioned medium (CM) was prepared by culturing C26 and 4T1 cells to 90% confluency, washing with PBS, and incubating in serum‐free DMEM for 48 h. The medium was then centrifuged to remove cell debris [16].

### 2.3 Puromycin Incorporation Assay

The Surface Sensing of Translation assay was performed to measure protein synthesis. Puromycin was added to the DM 1‐h before harvesting the C2C12 myotubes. Puromycin incorporation into the total protein was analysed by IB.

### 2.4 Myotube Morphology Analysis

Myoblast differentiation was assessed by measuring myotube diameter and fusion index using MyHC ICC staining. The DM was changed daily for 5 days, and fully differentiated C2C12 myotubes were analysed. Images were acquired using a confocal microscope (LSM900, Carl Zeiss) and processed using the ImageJ software (NIH). The fusion index was calculated as the percentage of total nuclei in the myotubes.

### 2.5 Gene Expression Analysis

Total RNA was extracted from mouse tibialis anterior (TA), HSkM, and C2C12 myotubes using QIAzol (Qiagen, Germany). RNA concentration was measured using a NanoDrop (ThermoFisher), and cDNA was synthesised using a reverse transcription system (Promega). mRNA expression was analysed by RT‐PCR (QuantStudio 3, ThermoFisher) using SYBR Green (#RT500, Enzynomics). The primer sequences used are listed in Table S1. Data were normalised to GAPDH expression, and fold‐changes were calculated using the ΔΔCt method.

### 2.6 Protein Analysis

Total protein was extracted from mouse TA or C2C12 myotube lysates using RIPA buffer supplemented with 1 mM Na_3_VO_4_, 1 mM PMSF, and 5 mM NaF. Protein concentrations were determined using the Bradford assay. Extracted proteins were subjected to SDS‐PAGE and transferred onto nitrocellulose membranes (Millipore). The membranes were then blocked in 5% BSA with TBST for 1 h, washed with TBST, and then incubated overnight with antibodies in a blocking buffer (5% BSA with TBST) at 4°C. Next, the membranes were washed with TBST to remove excess antibodies and incubated for 1 h with the HRP‐conjugated antibodies in blocking buffer. Protein expression was detected using enhanced chemiluminescence IB substrates (ThermoFisher) and visualised with a ChemiDoc imaging system (Solo6S; Vilber).

### 2.7 Cytosolic and Nuclear Protein Fractions

Cytoplasmic and nuclear proteins were extracted using CelLytic‐NuCLEAR Extraction Kit (#NXTRACT, Sigma) according to the manufacturer's instructions.

### 2.8 Immunofluorescence

For ICC, C2C12 cells were seeded in eight‐well chamber slides (SPL) and the differentiation of C2C12 myoblasts was initiated using DM. After a 5‐day differentiation, the cells were cultured under the indicated conditions. Subsequently, the cells were fixed in 4% paraformaldehyde for 5 min and blocked (5% BSA in PBS) for 30 min. The cells were then incubated overnight with anti‐MyHC or anti‐FoxO3a (5% BSA with PBS) at 4°C. After multiple washes with PBST, the cells were incubated with R‐phycoerythrin‐conjugated goat anti‐rabbit IgG (#P2771MP, 1:500, Invitrogen) or allophycocyanin‐conjugated goat anti‐mouse IgG (#A‐865, 1:500, Invitrogen) for 30 min at 25°C. The samples were then mounted onto slides using PBS with DAPI (#10236276001, Sigma) and visualised using a confocal microscope.

### 2.9 Co‐Immunoprecipitation Assay

Cell lysate proteins were incubated with rabbit anti‐FoxO3a or rabbit anti‐IgG for 24 h at 4°C. The immune complexes were captured using Protein A‐Sepharose (Amersham, UK) after 1 h of incubation. The precipitated immune complexes were washed five times with a wash buffer (25 mM HEPES, 5 mM EDTA, 1% Triton X‐100, 50 mM NaF, 150 mM NaCl, 10 mM PMSF) and denatured in an SDS sample buffer (125 mM Tris–HCl (pH 6.8), 20% glycerol, 4% SDS, 100 mM DTT, 0.1% bromophenol blue) by boiling at 95°C for 5 min.

### 2.10 siRNA Transfection

C2C12 myotubes were transfected with siAkt (#L‐040709‐00‐0010, Dharmacon), siFoxO3a (#L‐040728‐00‐0005, Dharmacon), or control siRNA (#sc‐37 007, SantaCruz) using Lipofectamine RNAiMax (Invitrogen) in Opti‐MEM. Four days post differentiation, the myotubes were transfected for 24 h and then switched to fresh DM.

### 2.11 Animal Experiments

Eight‐week‐old male and female BALB/C mice were purchased from Japan SLC (Hamamatsu) and housed in a controlled environment (temperature, 21°C–23°C; humidity, 50%–60%; 12‐h light:12‐h dark cycle). In previous studies, we used C26‐ and 4T1‐bearing mice as in vivo models for CAC [17, 18]. Mice were provided ad libitum access to water and a standard chow diet and acclimated for 1 week prior to the experiment. The male mice were randomly assigned to three groups (*n* = 12): sham, C26, and C26 + KIC. The female mice were randomly assigned to four groups (*n* = 10 each): sham, KIC, 4T1, and 4T1 + KIC. At 9 weeks of age, C26 or 4T1 cells (1 × 10^6^) were subcutaneously injected into both sides. Seven days after the injection, KIC (10 mg/kg) or PBS was administered intraperitoneally, daily for 21 days. On day 28, the mice were euthanised under isoflurane anaesthesia, and blood and tissue samples were analysed. Skeletal muscles including the tibialis anterior (TA), gastrocnemius (GCM), quadriceps (QA), soleus (SOL), and extensor digitorum longus (EDL) were carefully dissected from both hindlimbs. White adipose tissue (WAT) was collected from the epididymal region. Tumour weight was calculated using the formula: 0.52 × tumour length × tumour width [19]. Grip strength was measured using a calibrated grip strength tester (#JD‐A‐22, JeungDoBio), with mice gripping the bar using all four limbs. The results were expressed as the average of at least three repetitions.

### 2.12 Histology

Paraffin‐embedded muscle tissues were sectioned to a thickness of 8‐μm using a microtome and stained with haematoxylin and eosin for a quantitative tissue evaluation. Slides were imaged using a CKX53 microscope (Olympus, Japan), and cross‐sectional area (CSA) was measured using the ImageJ software, as previously reported [20].

### 2.13 Enzyme‐Linked Immunosorbent Assay (ELISA)

Cytokine levels in mouse serum and cell supernatants were measured using ELISA kits following the manufacturer's protocol: myostatin (#MBS8804274, MyBioSource), TNF‐α (#88‐7324‐88, Invitrogen), IFN‐γ (#MIF00, R&D‐Systems), and IL‐6 (#M6000B‐1, R&D‐Systems).

### 2.14 Statistical Analysis

Data were expressed as the mean ± standard error of the mean (SEM). Statistical differences between and among groups were evaluated using Student's *t*‐test and ANOVA followed by Tukey's post hoc test, respectively. Statistical analyses were conducted using Prism 5 (GraphPad), and significance levels of *p* < 0.05 were defined as *p < 0.05, **p < 0.01, and ***p < 0.001.

## 3 Results

### 3.1 Effect of KIC on Protein Turnover and Myostatin Expression

**FIGURE 1:**
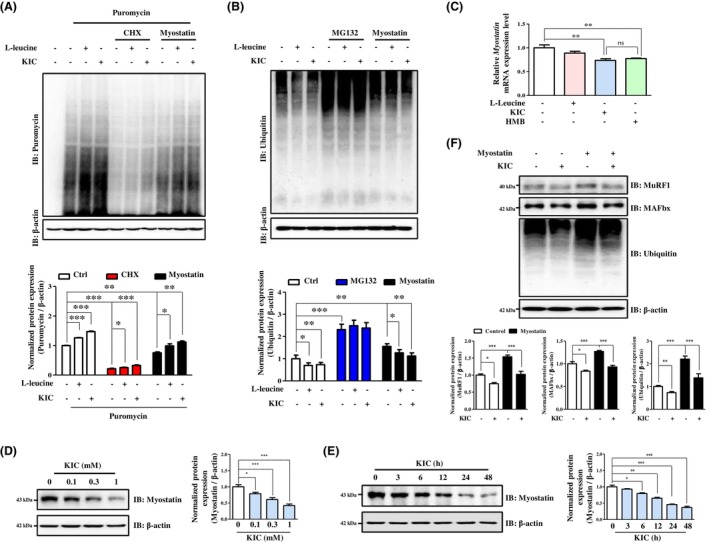
KIC regulates protein turnover and myostatin expression. (A) C2C12 myotubes were treated with CHX (10 μM) or myostatin (10 ng/mL), with or without l‐leucine (1 mM) or KIC (1 mM), for 24 h, followed by puromycin (10 μg/mL) treatment for 1 h. Protein synthesis was assessed by detecting puromycin incorporation via IB, normalized to β‐actin expression (*n* = 3). (B) C2C12 myotubes were treated with MG132 (10 μM) or myostatin (10 ng/mL), with or without l‐leucine (1 mM) or KIC (1 mM), for 24 h. Protein degradation was assessed by detecting ubiquitin‐conjugated proteins via IB, normalized to β‐actin expression (*n* = 3). (C) Relative *myostatin* mRNA levels in C2C12 myotubes treated with 1 mM l‐leucine metabolites (l‐leucine, KIC, and HMB) for 24 h were evaluated using RT‐PCR and normalized to those of GAPDH (*n* = 3). Myostatin protein expression (D) in C2C12 myotubes treated with KIC at the indicated doses (0.1–1 mM) for 48 h or (E) in C2C12 myotubes treated with KIC (1 mM) for the indicated times (3–48 h), analysed via IB and normalized to β‐actin expression (*n* = 3). (F) MuRF1 and MAFbx protein expression in myostatin (20 ng/mL) treated‐C2C12 myotubes, with or without KIC (1 mM) for 24 h, analysed via IB and normalized to β‐actin expression (*n* = 3). Results are expressed as mean ± SEM Statistical analysis was performed using one‐way ANOVA. *p < 0.05, **p < 0.01, ***p < 0.001 versus control. ANOVA, analysis of variance; CHX, cycloheximide; HMB, β‐hydroxy‐β‐methylbutyrate; IB, immunoblot; KIC, alpha‐ketoisocaproate; RT‐PCR, real‐time polymerase chain reaction; SEM, standard error of the mean.

We evaluated the cytotoxicity of l‐leucine metabolites (0.01–10 mM) in human and mouse skeletal muscle cells after 72 h of treatment. KIC maintained 50% viability at a higher concentration (C2C12: 4.68 mM; HSkM: 6.73 mM) than HMB (C2C12: 3.11 mM; HSkM: 3.24 mM) (Figure S1a). Therefore, the maximum treatment concentration was set as 1 mM. Myostatin disrupts protein metabolism in skeletal muscle [21]. To compare the effects of KIC and l‐leucine on protein turnover, we assessed puromycin incorporation and the expression of ubiquitin‐conjugated proteins as markers of protein synthesis and degradation, respectively. Myostatin treatment decreased puromycin incorporation and increased polyubiquitination, whereas KIC significantly restored these changes more effectively than did l‐leucine (Figure 1A,B, and Figure S1b,c). These findings indicate that KIC is more efficient than l‐leucine at counteracting myostatin‐mediated alterations in protein synthesis and degradation. Next, we investigated the effects of l‐leucine metabolites on muscle atrophy–related responses, such as altered myostatin expression. KIC and HMB significantly reduced luciferase activity compared with that with l‐leucine (Figure S1d). Both KIC and HMB markedly decreased myostatin mRNA levels, with no significant differences between the two treatments in either the mouse or human muscle cells (Figure 1C and Figure S1e). Consistently, KIC treatment reduced the myostatin protein levels in C2C12 myotubes (Figure 1D,E). Next, we examined whether KIC, which suppresses myostatin expression, inhibits the UPS in a myostatin‐dependent manner [6]. We indicate that KIC not only reduced polyubiquitination but also suppressed MuRF1 and MAFbx expression in a myostatin‐dependent manner (Figure 1F). These findings show that KIC may mitigate skeletal muscle atrophy by enhancing protein turnover and suppressing myostatin expression more effectively than l‐leucine.

### 3.2 KIC Rescues Myotube Atrophy Under CM‐Treated CAC‐Mimetic in Vitro Conditions by Inhibiting Myostatin

**FIGURE 2:**
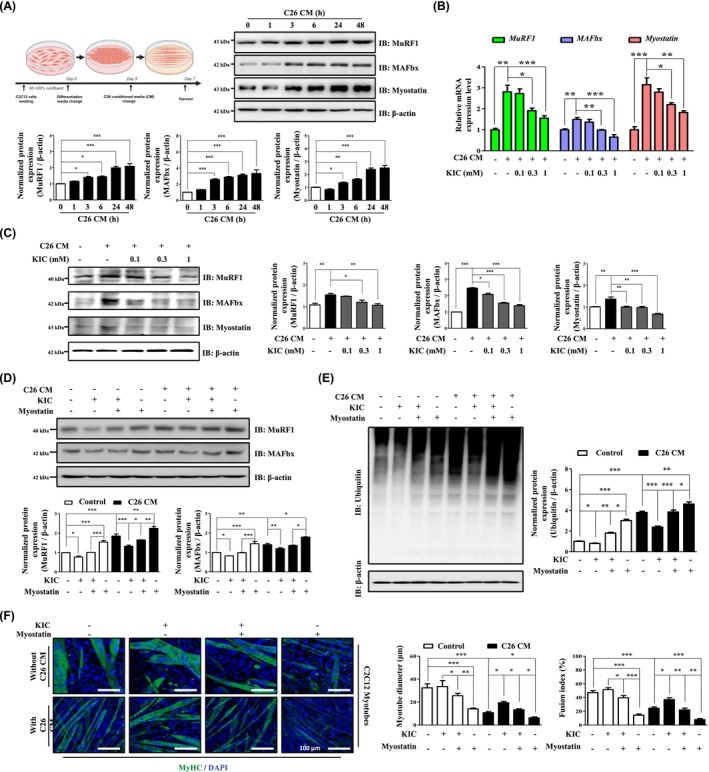
KIC inhibits myotube atrophy in C26‐CM‐treated C2C12 myotubes. (A) Experimental scheme for the CAC mimetic in vitro study. MuRF1, MAFbx, and myostatin protein expression in C2C12 myotubes cultured with DM containing 30% C26‐CM for the indicated times (1–48 h) was analysed using IB and normalized to β‐actin expression (*n* = 3). (B–C) C2C12 myotubes were incubated in DM containing 30% C26‐CM with or without KIC (0.1–1 mM) for 48 h. (B) Relative mRNA levels of *MuRF1*, *MAFbx*, and *myostatin* were assessed using RT‐PCR and normalized to those of *GAPDH* (*n* = 3). (C) Protein expression of MuRF1, MAFbx, and myostatin was analysed using IB and normalized to that of β‐actin (*n* = 3). (D–F) C2C12 myotubes were pretreated with myostatin (20 ng/mL) for 1 h, then incubated in DM containing 30% C26‐CM with or without KIC (0.3 mM) for 48 h. (D) MuRF1 and MAFbx protein expression was analysed using IB and normalized to that of β‐actin (*n* = 3). (E) Protein degradation was evaluated by detecting ubiquitin‐conjugated proteins using IB, normalized to β‐actin expression (n = 3). (F) MyHC ICC and morphological analysis of C2C12 myotubes. MyHC (green) and DAPI (blue) were used for visualisation. The myotube diameter was quantified using ImageJ (*n* = 50). The fusion index was calculated as the percentage of nuclei within MyHC‐positive myotubes (*n* = 5). The results are expressed as the mean ± SEM Statistical analysis was performed using one‐way ANOVA. *p < 0.05, **p < 0.01, ***p < 0.001 versus control. Scale bar, 100 μm. ANOVA, analysis of variance; CM, conditioned medium; DM, differentiation medium; IB, immunoblot; ICC, immunocytochemistry; KIC, alpha‐ketoisocaproate; RT‐PCR, real‐time polymerase chain reaction; SEM, standard error of the mean.

As KIC inhibits myotube atrophy in a myostatin‐dependent manner, we further investigated its potential to attenuate myotube atrophy under CAC‐mimetic conditions by utilising CM from C26 and 4T1 cells in vitro [16, 22]. Analysis of inflammatory cytokines in the CM revealed a significant increase in the levels of TNF‐α, IFN‐γ, and IL‐6 in both C26‐ and 4T1‐derived CM (Figure S2a). Treatment of C2C12 myotubes with C26‐CM significantly increased the expression of MuRF1, MAFbx, and myostatin (Figure 2A). Similarly, treatment with 4T1‐CM increased the myostatin protein levels (Figure S2b). These results indicated that the inflammatory environment in CAC‐mimetic conditions promotes upregulation of key atrophy‐related factors. We evaluated muscle atrophy‐related molecules in C2C12 myotubes treated with C26‐CM and found that KIC treatment significantly reduced both promoter‐level and mRNA expression (*MuRF1*, *MAFbx*, and *myostatin*) as well as protein levels (MuRF1, MAFbx, and myostatin) (Figures 2B,C and Figure S2c). A similar reduction was observed in 4T1‐CM‐treated C2C12 myotubes (Figure S2d,e). Next, we determined whether KIC could inhibit muscle atrophy‐related factors via a myostatin‐mediated mechanism under CAC‐mimetic conditions. The results showed that KIC significantly reduced MuRF1 and MAFbx expression and polyubiquitination in a myostatin‐dependent manner in C26‐CM‐treated C2C12 myotubes (Figure 2D,E). KIC treatment consistently restored C2C12 myotube diameter and fusion index in a myostatin‐dependent manner in C26‐CM‐treated atrophy (Figure 2F). Furthermore, KIC inhibited the reduction in the myotube diameter in 4T1‐CM‐treated C2C12 myotubes (Figure S2f). Overall, these findings demonstrate that KIC can inhibit myotube atrophy under CAC‐mimetic in vitro conditions.

### 3.3 MCT1‐2‐Mediated KIC Inhibits CM‐Treated Myotube Atrophy

**FIGURE 3:**
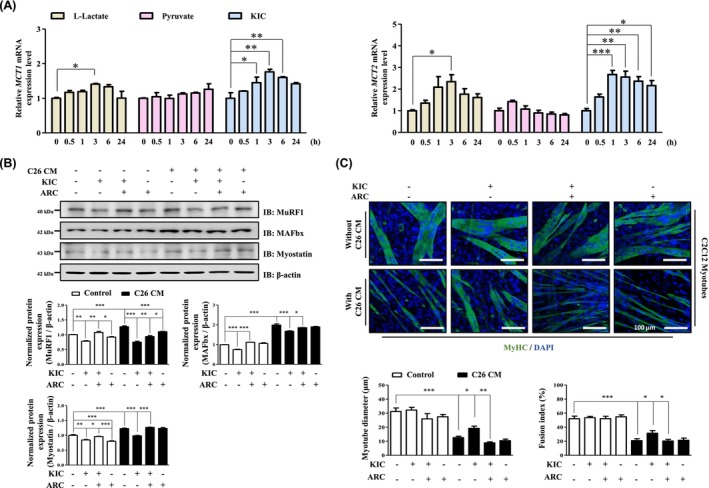
KIC inhibits myotube atrophy by mediating MCT1‐2 in C26‐CM‐treated C2C12 myotubes. (A) Comparison of the relative mRNA expression of *MCT1* and *MCT2* in C2C12 myotubes treated with 1 mM ketone bodies (l‐lactate, pyruvate, and KIC) for the indicated times (0.5–24 h). mRNA expression was evaluated by RT‐PCR and normalized to that of GAPDH (*n* = 3). (B–C) C2C12 myotubes were pretreated with ARC (100 nM) for 1 h, then incubated with DM containing 30% C26‐CM with or without KIC (0.3 mM) for 48 h. (B) Protein expression of MuRF1, MAFbx, and myostatin was evaluated using immunoblotting and normalized using β‐actin expression (*n* = 3). (C) MyHC ICC and morphological analysis of C2C12 myotubes. MyHC (green) and DAPI (blue) were used for visualisation. The myotube diameter was quantified using ImageJ (*n* = 50). The fusion index was calculated as the percentage of nuclei within MyHC‐positive myotubes (*n* = 5). Results are expressed as the mean ± SEM Statistical analysis was performed using one‐way ANOVA. *p < 0.05, **p < 0.01, and ***p < 0.001 versus control. Scale bar, 100 μm. ANOVA, analysis of variance; ARC, AR‐C155858; CM, conditioned media; DM, differentiation media; IB, immunoblot; ICC, immunocytochemistry; KIC, alpha‐ketoisocaproate; RT‐PCR, real‐time polymerase chain reaction; SEM, standard error of the mean.

Next, we investigated the transport proteins associated with KIC in skeletal muscle cells, hypothesising that KIC may influence skeletal muscles via monocarboxylate transporters (MCT), which mediate ketone body transport [23]. Previous studies have shown that MCT1 and MCT2 facilitate the uptake of ketone bodies, lactate, and pyruvate in skeletal muscles, particularly under exercise or metabolic stress, where their expression is also regulated by lactate and pyruvate [24]. Based on this, we examined whether KIC modulates MCT1 and MCT2 expression at the mRNA level. Our results showed that KIC treatment significantly increased the mRNA expression of *MCT1* and *MCT2* (Figure 3A). The MCT1‐2 inhibitor (ARC) was used to investigate whether KIC, which modulates MCT1‐2 expression, could mediate the suppression of myotube atrophy through MCT1‐2. The results showed that KIC treatment improved the fusion index and myotube diameter of C26‐CM‐treated C2C12 myotubes and reduced the expression of muscle atrophy‐related molecules. However, these effects were blocked by ARC (Figure 3B,C and Figure S3a). Similarly, KIC reduced the expression of muscle atrophy‐related molecules in 4T1‐CM‐treated C2C12 myotubes; however, this effect was also inhibited by ARC (Figure S3b). These findings suggest that skeletal muscle cells can mediate the effects of KIC via MCT1‐2.

### 3.4 KIC Inhibits CM‐Treated Myotube Atrophy via the Akt–FoxO3a Pathway

**FIGURE 4:**
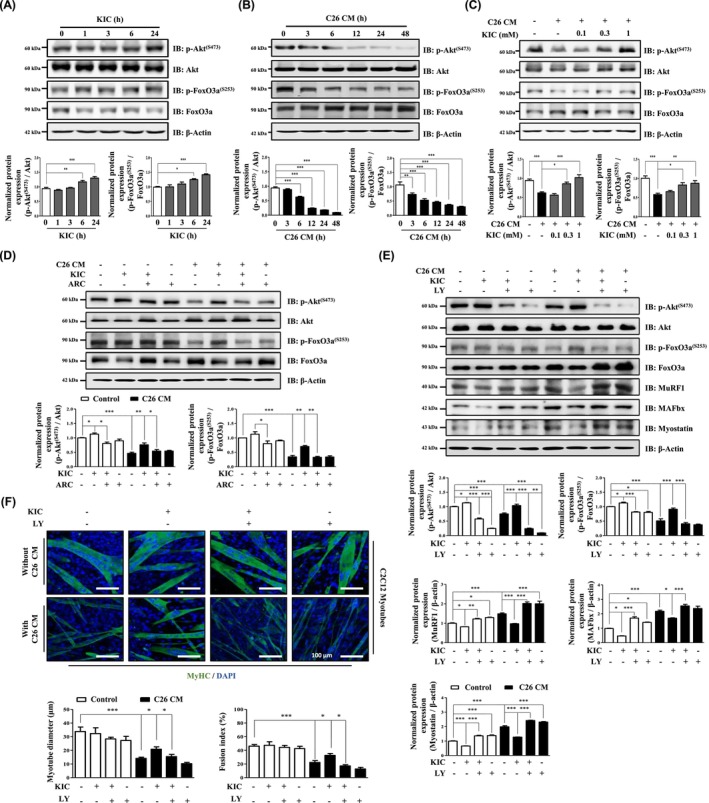
KIC phosphorylates Akt–FoxO3a in C2C12 myotubes. (A) Protein expression of p‐Akt^(ser473)^, Akt, p‐FoxO3a^(ser253)^, and FoxO3a in C2C12 myotubes cultured in DM containing KIC (0.3 mM) for the indicated times (1–24 h). (B) Protein expression of p‐Akt^(ser473)^, Akt, p‐FoxO3a^(ser253)^, and FoxO3a in C2C12 myotubes cultured in DM containing 30% C26‐CM for the indicated times (3–48 h). (C) Protein expression of p‐Akt^(ser473)^, Akt, p‐FoxO3a^(ser253)^, and FoxO3a in C2C12 myotubes were incubated in DM containing 30% C26‐CM with or without KIC (0.1–1 mM) for 48 h. (D) C2C12 myotubes were pretreated with ARC (100 nM) for 1 h, then incubated with DM containing 30% C26‐CM with or without KIC (0.3 mM) for 48 h. Protein expression of p‐Akt^(ser473)^, Akt, p‐FoxO3a^(ser253)^, and FoxO3a was evaluated using IB and normalized to that of Akt and FoxO3a (*n* = 3). (E–F) C2C12 myotubes were pretreated with LY (20 μM) for 1 h, then incubated with DM containing 30% C26‐CM with or without KIC (0.3 mM) for 48 h. (E) Protein expression of p‐Akt^(ser473)^, Akt, p‐FoxO3a^(ser253)^, FoxO3a, MuRF1, MAFbx, and myostatin was evaluated using IB and normalized using Akt, FoxO3a, and β‐actin (*n* = 3). (F) MyHC ICC and morphological analysis of C2C12 myotubes. MyHC (green) and DAPI (blue) were used for visualisation. Myotube diameter was quantified using ImageJ (*n* = 50). The fusion index was calculated as the percentage of nuclei within MyHC‐positive myotubes (*n* = 5). Results are expressed as the mean ± SEM Statistical analysis was performed using one‐way ANOVA. *p < 0.05, **p < 0.01, and ***p < 0.001 versus control. Scale bar, 100 μm. ANOVA, analysis of variance; ARC, AR‐C155858; CM, conditioned media; DM, differentiation media; IB, immunoblot; ICC, immunocytochemistry; KIC, alpha‐ketoisocaproate; LY, LY294002; SEM, standard error of the mean.

We investigated the mechanism influencing the myostatin promoter, as described by Esposito et al. in 2021 [25], focusing on the transcription factor FoxO3a, which regulates myostatin expression, and its upstream signalling molecule, Akt. KIC treatment decreased FoxO3a expression while increasing Akt–FoxO3a phosphorylation (Figure 4A). In contrast, C26‐CM treatment led to increased FoxO3a expression while reducing Akt–FoxO3a phosphorylation (Figure 4B). Similarly, 4T1‐CM treatment significantly decreased Akt–FoxO3a phosphorylation in C2C12 myotubes (Figure S4a). KIC treatment reversed these effects by increasing Akt–FoxO3a phosphorylation while decreasing FoxO3a expression in C26‐CM‐ and 4T1‐CM‐treated C2C12 myotubes (Figure 4C and Figure S4b). To determine whether MCT1‐2‐mediated transport of KIC is involved in restoring Akt–FoxO3a phosphorylation, we inhibited MCT1‐2. Our results showed that MCT1‐2 inhibition blocked the effects of KIC on Akt–FoxO3a phosphorylation and FoxO3a expression (Figure 4D). Additionally, we assessed whether the effects of KIC on myotube atrophy in CM‐treated C2C12 myotubes were Akt‐dependent, using LY, a PI3K/Akt inhibitor. KIC treatment reduced the expression of atrophy‐related molecules induced by C26‐CM and 4T1‐CM in C2C12 myotubes; this effect was blocked by LY treatment (Figure 4E and Figure S4c,d). Furthermore, KIC treatment improved the fusion index and myotube diameters in C26‐CM‐treated C2C12 myotubes, but this effect was also blocked by LY (Figures 4F). These results suggest that KIC influences Akt activation in skeletal muscles through MCT1‐2 transporters, thereby alleviating myotube atrophy under CAC‐mimetic conditions.

### 3.5 KIC Inhibits Myotube Atrophy by FoxO3a Inactivation in CM‐Treated C2C12 Myotubes

**FIGURE 5:**
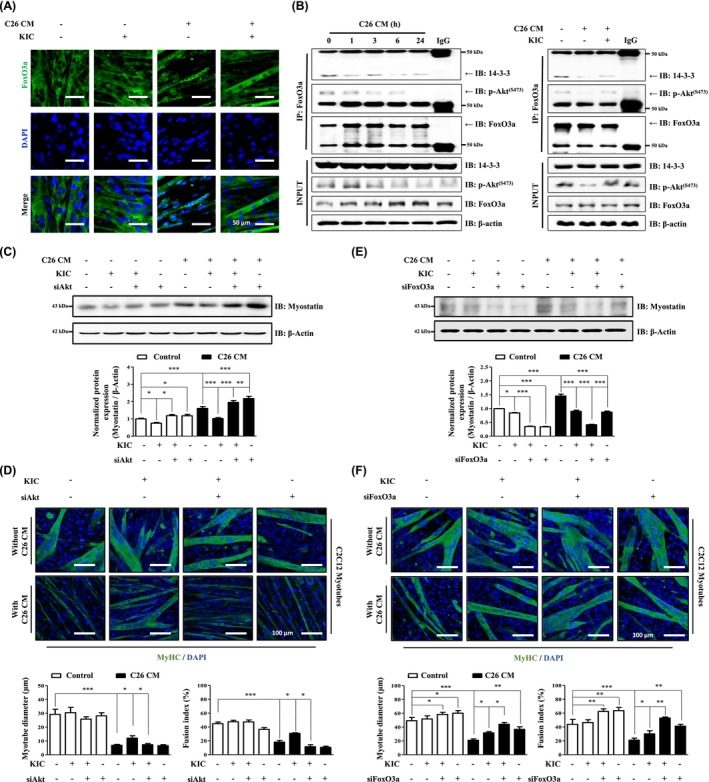
KIC inhibits myotube atrophy by enhancing p‐Akt and FoxO3a interaction and inhibiting FoxO3a translocation in C26‐CM‐treated C2C12 myotubes. (A) Localisation of FoxO3a was assessed using ICC in C2C12 myotubes incubated in DM containing 30% C26‐CM, with or without KIC (0.3 mM) for 48 h. Cells were stained for FoxO3a (green) and DAPI (blue) and visualized using confocal microscopy. (B) Protein–protein interaction between 14‐3‐3 proteins, p‐Akt^(ser473)^, and FoxO3a in C2C12 myotubes cultured in DM containing 30% C26‐CM for the indicated times (1–24 h) or in DM containing 30% C26‐CM with or without KIC (0.3 mM) for 24 h. Protein–protein interactions were evaluated by Co‐IP performed using an anti‐FoxO3a antibody (*n* = 3). (C–F) C2C12 myotubes were transfected with Akt or FoxO3a siRNA (100 nM) or control siRNA (100 nM) and incubated for 24 h, followed by treatment with DM containing 30% C26‐CM, with or without KIC (0.3 mM) for 48 h. (C, E) Myostatin protein expression was evaluated by IB and normalized to that of β‐actin (*n* = 3). (D, F) MyHC ICC and morphological analysis were performed using MyHC (green) and DAPI (blue) for visualisation. Myotube diameter was quantified using ImageJ (*n* = 50), and the fusion index was calculated as the percentage of nuclei within MyHC‐positive myotubes (*n* = 5). Results are expressed as the mean ± SEM Statistical analysis was performed using one‐way ANOVA. *p < 0.05, **p < 0.01, and ***p < 0.001 versus control. Scale bar in (B) = 50 μm, and in (D, F) = 100 μm. ANOVA, analysis of variance; CM, conditioned media; Co‐IP, co‐immunoprecipitation; DM, differentiation media; IB, immunoblot; ICC, immunocytochemistry; KIC, alpha‐ketoisocaproate; RT‐PCR, real‐time polymerase chain reaction; SEM, standard error of the mean.

To clarify the role of KIC in FoxO3a activation, we examined its effect on the translocation of FoxO3a to the nucleus, where it acts as a transcription factor for myostatin. Nuclear and cytoplasmic fractions were isolated to assess the protein expression levels. Treatment with C26‐CM and 4T1‐CM increased FoxO3a expression in the nucleus, whereas KIC treatment reduced the nuclear FoxO3a levels (Figure S5a,b). FoxO3a localisation was further analysed using confocal microscopy. Under C26‐CM and 4T1‐CM treatment, FoxO3a localisation was nucleus‐specific and was significantly reduced following co‐treatment with KIC (Figure 5A and Figure S5c). To further understand the effect of KIC on the interaction between Akt and FoxO3a, we investigated this interaction in CM‐treated C2C12 myotubes. Because Akt activation induces FoxO3a phosphorylation, we investigated the interaction between FoxO3a and 14‐3‐3 proteins, which are regulatory proteins that bind to specific Ser/Thr phosphorylation motifs in target proteins. We performed Co‐IP of FoxO3a and p‐Akt using lysates from C2C12 myotubes treated with C26‐CM and 4T1‐CM. Our results showed that the interaction between the 14‐3‐3 proteins, p‐Akt, and FoxO3a was significantly reduced after 6 and 24 h of treatment. To investigate whether KIC could reverse this effect, we co‐treated cells with KIC and CM for 24 h. The results showed that KIC treatment restored the interactions between FoxO3a, 14‐3‐3 proteins, and p‐Akt, which had been reduced by C26‐CM and 4T1‐CM (Figure 5B and Figure S5d). As KIC treatment enhanced the interaction between p‐Akt and FoxO3a, we further investigated the role of Akt and FoxO3a signalling in regulating myostatin expression. Akt knockdown in C2C12 myotubes treated with C26‐CM significantly increased myostatin expression (Figure 5C and Figure S5e) and reduced the fusion index and myotube diameter (Figure 5D). In contrast, siRNA‐mediated knockdown of FoxO3a decreased myostatin expression and improved the fusion index and myotube diameter in C26‐CM‐treated C2C12 myotubes (Figure 5E,F, and Figure S5f). These findings indicate that KIC improves the interaction between FoxO3a and p‐Akt, thereby reducing the activation and translocation of FoxO3a to the nucleus, suggesting that p‐Akt and FoxO3a are upstream molecules that regulate myostatin expression.

### 3.6 KIC Attenuates CAC Through the Akt–FoxO3a–Myostatin Pathway in C26‐Injected Mice

**FIGURE 6:**
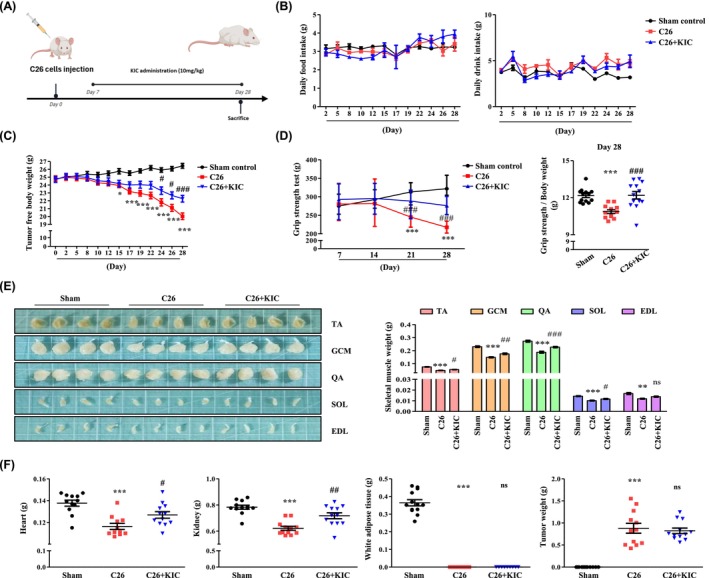
KIC administration prevents CAC in C26 cell‐injected mice. (A) Scheme of the experimental schedule for the CAC animal study. (B) Daily food and drink intake. (C) Tumour‐free body weight. (D) Grip strength testing during days 0–28. Each result is expressed as the mean ± SEM of *n* = 12 mice in each group. Two‐way ANOVA was used to determine statistical significance. *p < 0.05, **p < 0.01, and ***p < 0.001 versus sham; #*p* < 0.05, ##*p* < 0.01, and ###*p* < 0.001 versus C26. (E) Skeletal muscle (TA, GCM, QA, SOL, and EDL) morphology and skeletal muscle weight was evaluated at the time of sacrifice (*n* = 12 per group). (F) The heart, kidney, white adipose tissue, and tumour weights were measured at the time of sacrifice (*n* = 12 per group). Results are expressed as the mean ± SEM. One‐way ANOVA was used to determine statistical significance. *p < 0.05, **p < 0.01, and ***p < 0.001 versus sham; #*p* < 0.05, ##*p* < 0.01, and ###*p* < 0.001 versus C26. Sham: sham mice; C26: C26‐injected mice; C26 + KIC: C26‐injected mice treated with KIC (10 mg/kg) intraperitoneally. ANOVA, analysis of variance; CAC, cancer‐associated cachexia; EDL, extensor digitorum longus; GCM, gastrocnemius; KIC, alpha‐ketoisocaproate; QA, quadriceps; SEM, standard error of the mean; SOL, soleus; TA, tibialis anterior.

**FIGURE 7:**
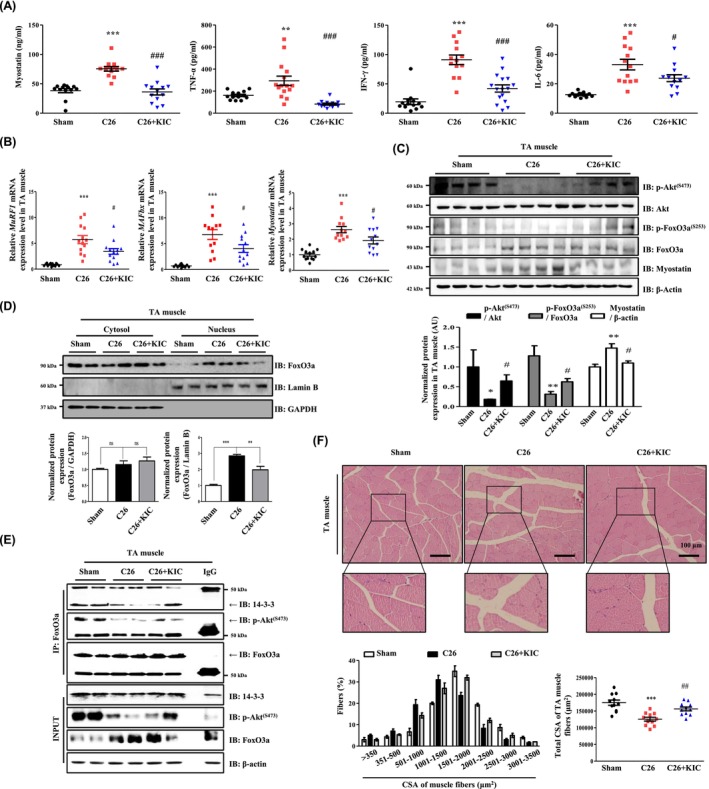
KIC administration prevents CAC via the Akt–FoxO3 pathway in C26 cell‐injected mice. (A) Myostatin, TNF‐α, IFN‐γ, and IL‐6 levels in the serum were analysed using ELISA (*n* = 12 per group). (B) Comparison of the relative mRNA expression of *MuRF1*, *MAFbx*, and *Myostatin* in TA muscle evaluated using RT‐PCR and normalized using GAPDH expression (*n* = 12 per group). (C) Protein expression of p‐Akt^(ser473)^, Akt, p‐FoxO3a^(ser253)^, FoxO3a, and myostatin in TA muscle evaluated using IB was normalized using Akt, FoxO3a, and β‐actin expression (*n* = 3). (D) The lysate of TA muscle was fractionated into nuclear and cytoplasmic fractions. FoxO3a protein expression evaluated using IB was normalized using the cytoplasmic protein GAPDH and the nuclear protein Lamin B (*n* = 3). (E) Protein–protein interaction between p‐Akt^(ser473)^ and FoxO3a in TA muscle. Protein–protein interactions were evaluated by Co‐IP performed using an anti‐FoxO3a antibody (*n* = 3). (F) Representative HE‐stained images of CSA in TA muscle show the fibre size distribution and average fibre CSA (*n* = 12 per group). Results are expressed as the mean ± SEM One‐way ANOVA was used to determine statistical significance. *p < 0.05, **p < 0.01, and ***p < 0.001 versus sham; #*p* < 0.05, ##*p* < 0.01, and ###*p* < 0.001 versus C26. Sham: sham mice; C26: C26‐injected mice; C26 + KIC: C26‐injected mice treated with KIC (10 mg/kg) intraperitoneally. Scale bar, 100 μm. ANOVA, analysis of variance; CAC, cancer‐associated cachexia; Co‐IP, co‐immunoprecipitation; CSA, cross‐sectional area; ELISA, enzyme‐linked solvent assay; HE, haematoxylin–eosin; IB, immunoblot; KIC, alpha‐ketoisocaproate; SEM, standard error of the mean; TA, tibialis anterior.

To validate the in vivo efficacy of KIC, we evaluated its effects on CAC induced by digestive cancer cells [17]. CAC was induced by subcutaneous injection of C26 cells into both flanks. KIC (10 mg/kg) was administered intraperitoneally from days 7 to 28, as this route ensures systemic exposure due to its high intestinal absorption [26]. The mice were sacrificed on day 28 for analysis (Figure 6A). We examined CAC symptoms including food intake, body weight, muscle mass, and strength. Food and drink intake showed no significant differences (Figure 6B); however, tumour‐free body weight significantly declined by day 14 of the CAC experiment (*p* < 0.05) and increased on day 14 after KIC administration (day‐21 of the experiment) (*p* < 0.05) (Figure 6C). KIC significantly improved grip strength, normalised to body weight by day 28, reaching levels comparable with those in the sham group (*p* < 0.001) (Figure 6D). Muscle mass of the TA, GCM, QA, and SOL muscles also significantly increased with KIC treatment (Figure 6E). Additionally, KIC increased the weights of the skeletal muscle, heart (*p* < 0.05), and kidney (*p* < 0.01), whereas the weights of WAT and tumours remained unchanged (Figure 6F). These results showed that KIC administration had no effect on tumour growth but inhibited skeletal muscle loss. Subsequently, we explored the molecular mechanisms underlying KIC action in C26‐injected mice. Further, we observed a significant decrease in the levels of myostatin (*p* < 0.001), TNF‐α (*p* < 0.001), IFN‐γ (*p* < 0.001), and IL‐6 (*p* < 0.05) after KIC administration in C26‐injected mice (Figure 7A). Furthermore, KIC treatment reduced myostatin, MuRF1, and MAFbx transcript levels in the TA muscle (*p* < 0.05) (Figure 7B). Next, we investigated the effect of KIC on myostatin expression via Akt–FoxO3a phosphorylation. Protein expression analysis of the TA muscle revealed elevated myostatin expression and reduced Akt–FoxO3a phosphorylation in C26‐injected mice compared with that in the sham group. KIC administration reversed these changes (Figure 7C). To further examine FoxO3a activation, we isolated the nuclear and cytoplasmic fractions from TA muscles. FoxO3a expression was significantly higher in the nucleus of C26‐injected mice, and KIC treatment reversed this effect (Figure 7D). Additionally, Co‐IP analysis showed that KIC restored the interactions between 14‐3‐3 proteins, p‐Akt, and FoxO3a, which were reduced in C26‐injected mice (Figure 7E). Moreover, histological analysis of the TA muscle fibres to examine the CSA corroborated our findings (Figure 7F). These findings show that KIC alleviates CAC‐induced muscle atrophy in the C26‐bearing mouse model by enhancing the interaction between FoxO3a and p‐Akt, while suppressing myostatin expression.

### 3.7 KIC Attenuates CAC Symptoms in 4T1‐Injected Mice

**FIGURE 8:**
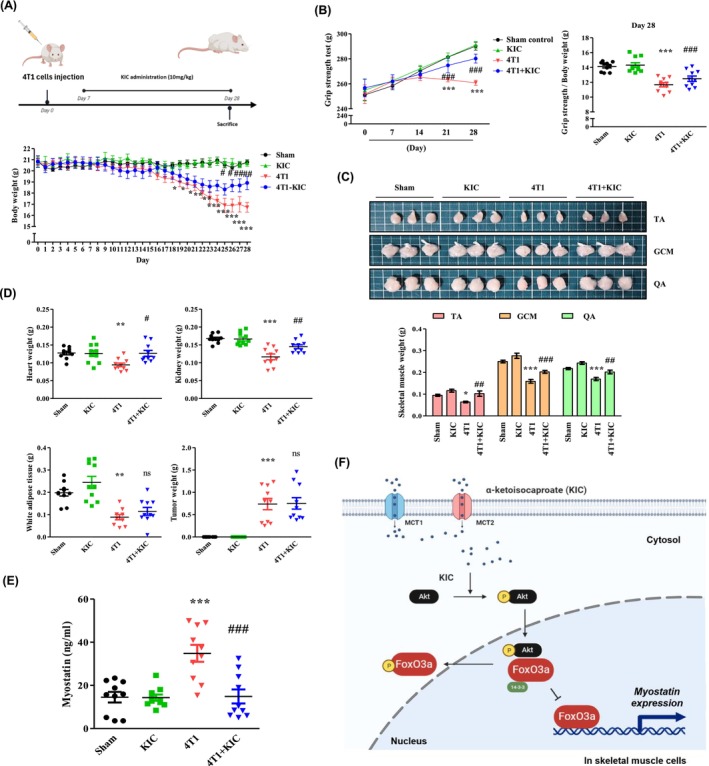
KIC administration prevents CAC in 4T1 cell‐injected mice. (A) Scheme of the experimental schedule for the CAC animal study and tumour‐free body weight determination. (B) Grip strength testing during days 0–28. Each result was expressed as the mean ± SEM of *n* = 10 mice in each group. Two‐way ANOVA was used to determine statistical significance. *p < 0.05, **p < 0.01, and ***p < 0.001 versus sham; #*p* < 0.05, ##*p* < 0.01, and ###*p* < 0.001 versus 4T1. (C) Skeletal muscle (TA, GCM, and QA) morphology and skeletal muscle weight of mice were evaluated at the time of sacrifice (*n* = 10 per group). (D) The heart, kidney, white adipose tissue, and tumour weights were measured at the time of sacrifice (*n* = 10 per group). (E) Myostatin levels in serum were analysed using ELISA (*n* = 10 per group). One‐way ANOVA was used to determine statistical significance. *p < 0.05, **p < 0.01, and ***p < 0.001 versus sham; #*p* < 0.05, ##*p* < 0.01, and ###*p* < 0.001 versus 4T1. (F) Schematic diagram of the mechanism of action of KIC: KIC is transported into skeletal muscle cells via MCT1–2 and activates Akt. KIC inhibits FoxO3a activation in an Akt‐dependent manner, leading to the export of FoxO3a from the nucleus. Consequently, sequestration of FoxO3a from the nucleus alleviates cancer cachexia by inhibiting myostatin. Sham: sham mice; KIC: mice treated with KIC (10 mg/kg) intraperitoneally. 4 T1: 4T1‐injected mice; 4T1 + KIC: 4T1‐injected mice treated with KIC (10 mg/kg) intraperitoneally. ANOVA, analysis of variance; CAC, cancer‐associated cachexia; ELISA, enzyme‐linked solvent assay; GCM, gastrocnemius; KIC, alpha‐ketoisocaproate; QA, quadriceps; SEM, standard error of the mean; TA, tibialis anterior.

We extended our investigations to a mice injected with 4T1, a non‐gastrointestinal cancer cell line (breast cancer cell), to assess the reproducibility of the in vivo effects of KIC observed in the C26 model [18]. We assessed CAC symptoms in 4T1‐injected mice. The tumour‐free body weights remained unchanged in the KIC‐treated and Sham groups during the 28‐day experiment. However, 4T1‐injected mice showed significant weight loss from day 7, which KIC treatment reversed by day 23 (*p* < 0.05) (Figure 8A). KIC also improved grip strength from day 14 (*p* < 0.001) (Figure 8B) and increased the muscle mass of the TA, GCM, and QA (Figure 8C). Furthermore, KIC significantly increased the weights of the skeletal muscle, heart (*p* < 0.05), and kidney (*p* < 0.01), but did not affect the WAT or tumour weights (Figure 8D). To examine whether the observed effects were mediated by myostatin inhibition, we measured serum myostatin levels and found that KIC significantly reduced the elevated levels in 4T1‐injected mice, restoring them to those of the sham group (*p* < 0.001) (Figure 8E). To further validate the efficacy of KIC, we evaluated myostatin expression in C2C12 myotubes exposed to tumour‐derived conditioned media (TCM) obtained from tumours excised from C26‐ and 4T1‐injected mice, thereby mimicking the cachectic tumour microenvironment in vitro (Figure S6a). TCM treatment upregulated myostatin expression and suppressed Akt–FoxO3a phosphorylation, whereas these effects were reversed by KIC, suggesting that KIC may regulate myostatin expression via the Akt/FoxO3a signalling pathway (Figure S6b,c). KIC treatment also restored both the myotube diameter and fusion index in TCM‐induced atrophic C2C12 myotubes (Figure S6d). These findings collectively indicate that KIC alleviates CAC‐induced muscle atrophy both in vivo and in vitro.

## 4 Discussion

Currently, appetite stimulants, such as anamorelin and megestrol, are recommended for CAC treatment, but these have limitations. For instance, anamorelin may not be suitable for patients with hyperglycaemia, and megestrol may cause unintended side effects such as sexual dysfunction [27, 28]. To avoid these limitations, we investigated l‐leucine metabolites. We elucidated the role of KIC in regulating myostatin expression in skeletal muscles in a C26‐ and 4T1‐induced model. Several studies have used C26‐ and 4T1‐injected mice, as well as C26‐CM and 4T1‐CM, as suitable experimental models for studying CAC‐mimicking conditions characterised by increased myostatin expression [16, 17, 18, 22]. Strategies targeting myostatin can inhibit skeletal muscle decline in experimental models of CAC‐mimetic conditions. In our study, we found that KIC, a metabolite of l‐leucine, inhibited muscle atrophy by targeting myostatin in CAC models. We further demonstrated that KIC improved protein turnover and enhanced muscle mass through myostatin‐dependent regulation. In addition to promoting protein turnover, KIC suppressed the expression of MuRF1 and MAFbx, key components involved in muscle protein degradation, in a myostatin‐dependent manner.

Previous studies have shown that both l‐leucine and its metabolites can phosphorylate Akt [15]. Based on these results, we show that KIC, as a therapeutic agent, alleviated muscle atrophy in a CAC model through Akt phosphorylation. We demonstrated that KIC induced the phosphorylation of Akt‐FoxO3a in vivo and in vitro. Inhibition of myostatin expression was associated with FoxO3a activation in an Akt‐dependent manner. Several studies support our results, showing a relationship between Akt and myostatin. For example, in diabetic muscle atrophy, increased myostatin expression is associated with changes in DNA damage/repair and ATP production pathways along with decreased Akt/mTOR/FoxO signalling, which deteriorates skeletal muscle degradation [29]. Overexpression of myostatin in mice affects the Akt/mTOR pathway, inhibiting the decreased phosphorylation of the ribosomal proteins S6 and 4E‐BP1 [30]. Reduced mTOR signalling exacerbates muscle atrophy in CAC, whereas activation of Akt–mTORC1 signalling in the skeletal muscle reverses muscle loss and restores muscle function [31]. A strategy to phosphorylate the Akt–FoxO3a signalling pathway targeting myostatin, which affects muscle loss, and Akt, which is involved in muscle protein synthesis, could thus be an appropriate treatment target for CAC. Our results demonstrate that KIC induces Akt–FoxO3a phosphorylation through MCT1‐2. Phosphorylated Akt inhibits activation of the transcription factor FoxO3a, which in turn suppresses myostatin expression and ultimately alleviates muscle atrophy under CAC conditions (Figure 8F). Interestingly, although KIC reduced myostatin expression, it did not induce muscle hypertrophy under normal conditions, suggesting that while KIC may inhibit muscle catabolism under cachectic conditions, its anabolic potential appears to be limited in non‐pathological states.

Our results confirm the potential role of MCT1‐2‐mediated KIC in skeletal muscle cells. Several studies have reported that KIC can mediate MCT1‐2 as a monocarboxylate. The lack of MCT1‐2 in the developing brain may result in KIC accumulation [32]. Although the MCT1‐2‐mediated response is controversial because it prolongs cancer cell survival, its role in skeletal muscle remains unclear [33]. Our results demonstrated that MCT1‐2‐mediated KIC improved muscle atrophy by inducing Akt–FoxO3a phosphorylation. The role of KIC in stimulating protein synthesis in CAC may involve mTOR regulation via Akt activation, but its effect on reversible transamination via l‐leucine remains to be demonstrated [34]. Previous studies have demonstrated that cardiac and skeletal muscle cells depleted or treated with exogenous branched‐chain keto acid (BCKA) or BCKA dehydrogenase exhibit defective insulin signalling, resulting in mTORC1 activation and protein translation [35]. Activation of mTOR signalling and reduction of reactive oxygen species production emphasises the significance of BCAA transaminase (BCAT) 1 expression in sustaining muscle cell growth [36]. Another study suggests that the use of l‐leucine metabolites improves insulin resistance in BCAT2‐depleted cells in skeletal muscle [37]. Our results show that KIC administration improves muscle atrophy in CAC without affecting tumour growth. Therefore, further research on the effects of KIC on cancer cells is necessary to understand its potential adverse effects.

Overall, we identified KIC as a potential therapeutic candidate for muscle atrophy conditions such as CAC. KIC supplementation enhanced muscle mass and function by modulating protein synthesis, skeletal muscle regeneration, and proteolysis. Studies have also demonstrated that taking KIC before acute physical activity led to a 10% increase in muscle work and a reduction in muscle fatigue during the early phase of exercise [38]. As a metabolite of leucine, KIC undergoes rapid metabolism in vivo, with approximately 97.8% conversion and a reported half‐life of 14.3 min [39, 40]. Given its potential for MCT‐mediated transport, its therapeutic application may be limited by its short half‐life and bioavailability. Additionally, our results showed that KIC administration increased kidney mass in vivo, possibly due to alterations in cellular metabolism or activation of anabolic signalling pathways. Consequently, further research is required to investigate potential off‐target effects or systemic adaptations, as well as to explore prodrug derivatives or sustained‐release formulations targeting MCT1‐2 in skeletal muscles to enhance pharmacodynamic properties and maximise therapeutic efficacy.

In summary, our study showed that KIC administration is required in CAC to confirm the direct Akt–FoxO3a relationship, which can trigger inhibition of myostatin expression. The evidence presented here demonstrates that KIC treatment effectively inhibits muscle atrophy in both C26‐ and 4T1‐injected mice and C26‐CM‐ and 4T1‐CM‐induced C2C12 myotubes, suggesting that KIC has potential as a therapeutic candidate for improving skeletal muscle protein degradation in CAC.

## Ethics Statement

The Institutional Animal Care and Use Committee of Korea University approved all animal experiments, which were conducted according to relevant guidelines and regulations (KOREA‐2022‐0113).

## Conflicts of Interest

The authors declare no conflicts of interest.

## Supporting information


**Data S1** Supplementary Information.

**Data S2** Supplementary Information.

**Data S3** Supplementary Figures.

**Table S1** Nucleotide sequences of primers and the operating condition of RT‐PCR.

## Data Availability

No datasets were generated or analysed during the current study.
