# Supplementary material for: Alpha‐Ketoisocaproate Attenuates Muscle Atrophy in Cancer Cachexia Models
Source: J Cachexia Sarcopenia Muscle. 2025 Aug 14;16(4):e70044. doi: 10.1002/jcsm.70044 (PMC12351804; doi:10.1002/jcsm.70044)
Supplement: Supplementary file 1 — Data S1 Supplementary Information. [file JCSM-16-e70044-s004.docx]

**Methods**

***Cell viability assay***

C2C12 and HSkM cells were seeded in 96-well plates and treated with L-leucine, KIC, or HMB (0.01–10 mM) for 24–72 h. After incubation with MTT (37°C, 4 h), the medium was discarded, formazan was eluted with DMSO, and absorbance was measured at 540 nm.

***Luciferase reporter assay***

Myostatin promoter activity was evaluated using a luciferase reporter in myostatin luciferase-expressing C2C12 cells prepared according to a previously established method.^1^

***Collection of tumor-derived CM***

Tumor-derived conditioned medium (TCM) was prepared based on previously reported tumor dissociation methods^2,3^. Briefly, tumor tissues were harvested on day 14 from BALB/c mice subcutaneously injected with C26 or 4T1 cancer cells (1×10⁶ cells). Excised tumors were rinsed with PBS, finely minced in serum-free RPMI1640 medium, and enzymatically digested with collagenase D and dispase (Roche) at 37°C for 30 min. The resulting cell suspension was passed through a 70-μm cell strainer, and the filtered cells were cultured in RPMI1640 medium supplemented with 10% FBS and penicillin-streptomycin. After several passages, cells at 90% confluence were washed with PBS and incubated in serum-free DMEM for 48 h. The collected medium was centrifuged to remove cellular debris and used as TCM.

**Refference**

1. Kang MJ, Moon JW, Lee JO, Kim JH, Jung EJ, Kim SJ, et al. Metformin induces muscle atrophy by transcriptional regulation of myostatin via HDAC6 and FoxO3a. *J Cachexia Sarcopenia Muscle* 2022;**13**:605-620.

2. Gorman S, Tosetto M, Lyng F, Howe O, Sheahan K, O'Donoghue D, et al. Radiation and chemotherapy bystander effects induce early genomic instability events: telomere shortening and bridge formation coupled with mitochondrial dysfunction. *Mutat Res* 2009;**669**:131-8.

3. Rodriguez de la Fuente L, Law AMK, Gallego-Ortega D, Valdes-Mora F. Tumor dissociation of highly viable cell suspensions for single-cell omic analyses in mouse models of breast cancer. *STAR Protoc* 2021;**2**:100841.
