# Supplementary material for: Alpha‐Ketoisocaproate Attenuates Muscle Atrophy in Cancer Cachexia Models
Source: J Cachexia Sarcopenia Muscle. 2025 Aug 14;16(4):e70044. doi: 10.1002/jcsm.70044 (PMC12351804; doi:10.1002/jcsm.70044)
Supplement: Supplementary file 2 — Data S2 Supplementary Information. [file JCSM-16-e70044-s002.docx]

**Fig. S1.** KIC regulates protein turnover and myostatin expression. (a) C2C12 and HSkM treated with KIC at the indicated doses (0.01–10 mM) for indicated times (24–72 h). C2C12 and HSkM treated with L-leucine metabolites (L-leucine, KIC, and HMB) at the indicated doses (0.01–10 mM) for 72 h. Cell viability was evaluated using the MTT assay (n = 3). (b) C2C12 myotubes were treated with L-leucine (0.1–1 mM) or KIC (0.1–1 mM) for 24 h, followed by puromycin (10 μg/ml) treatment for 1 h before harvest. Protein synthesis was assessed by detecting puromycin incorporation using IB and normalized to β-actin (n = 3). (c) C2C12 myotubes were treated with L-leucine (0.1–1 mM) or KIC (0.1–1 mM) for 24 h. Protein degradation was assessed by detecting ubiquitin-conjugated proteins using IB, normalized to β-actin (n = 3). (d) Luciferase activity in myostatin luciferase-expressing C2C12 cells treated with l-leucine metabolites at the indicated doses (0.01–1 mM) for 12 h was evaluated using the luciferase assay (n = 3). (e) Comparison of the relative mRNA expression of *myostatin* in HSkM treated with L-leucine metabolites (1 mM) for 24 h was evaluated using RT-PCR and normalized using GAPDH (n = 3). Each data points were expressed as mean ± SEM. Statistical analysis was performed using one-way ANOVA. *p < 0.05, **p < 0.01, and ***p < 0.001 versus control. ANOVA, analysis of variance; HMB, β-hydroxy-β-methylbutyrate; HSkM, human skeletal muscle cell; IB, immunoblotting; KIC, alpha-ketoisocaproate; RT-PCR, real-time polymerase chain reaction; SEM, standard error of the mean.

**Fig. S2.** KIC inhibits myotube atrophy in 4T1-CM-treated C2C12 myotubes. (a) Inflammatory cytokine (TNF-α, IFN-γ, and IL-6) levels in C26- and 4T1-CM were evaluated by ELISA (n =3). (b) Schema of experimental schedule for CAC mimetic *in vitro* study. Protein expression of myostatin in C2C12 myotubes cultured in DM containing 30% 4T1-CM at indicated times (1–48 h) was evaluated using IB and normalized using β-actin (n = 3). (c) Luciferase activity was measured in myostatin luciferase-expressing C2C12 cells cultured in DM containing 30% C26-CM or 4T1-CM, with or without KIC (0.3 mM) treatment, for 12 h using a luciferase assay (n = 3). (d-f) C2C12 myotubes were analyzed after incubation for 48 h in DM containing 30% 4T1-CM with or without KIC (0.3 mM). (d) Comparison of the relative mRNA expression of *MuRF1*, *MAFbx*, and *myostatin* was evaluated using RT-PCR and normalized using GAPDH (n = 3). (e) Protein expression of MuRF1, MAFbx, and myostatin was evaluated using IB and normalized using β-actin (n = 3). (f) MyHC ICC and myotube morphology analysis. MyHC (green) and DAPI (blue) were used for visualization. Myotube diameter was quantified using ImageJ (n = 50). The fusion index was calculated as the percentage of nuclei within MyHC-positive myotubes (n = 5). Each data points were expressed as mean ± SEM. Statistical analysis was performed using one-way ANOVA. *p < 0.05, **p < 0.01, and ***p < 0.001 versus control. Scale bar, 100 μm. ANOVA, analysis of variance; CM, conditioned media; DM, differentiation media; ELISA, enzyme-linked sorbent assay; IB, immunoblot; ICC, immunocytochemistry; KIC, alpha-ketoisocaproate; RT-PCR, real-time polymerase chain reaction; SEM, standard error of the mean; SF, Serum free media.

**Fig. S3.** KIC inhibits myotube atrophy via MCT1-2 in C26-CM or 4T1-CM-treated C2C12 myotubes. (a, b) C2C12 myotubes were pretreated with ARC (100 nM) for 1 h, then incubated with DM containing 30% CM with or without KIC (0.3 mM) for 48 h. Relative mRNA expression of *MuRF1*, *MAFbx*, and *myostatin* was analyzed using RT-PCR and normalized to GAPDH. (a) C26-CM treatment. (b) 4T1-CM treatment. Each data point represents triplicate measurements (n = 3). Statistical analysis was performed using one-way ANOVA. *p < 0.05, **p < 0.01, and ***p < 0.001 versus control. ANOVA, analysis of variance; ARC, AR-C155858; CM, conditioned media; DM, differentiation media; KIC, alpha-ketoisocaproate; RT-PCR, real-time polymerase chain reaction; SEM, standard error of the mean.

**Fig. S4.** KIC phosphorylates Akt–FoxO3a in C2C12 myotubes. (a) Protein expression of p-Akt^(ser473)^, Akt, p-FoxO3a^(ser253)^, and FoxO3a in C2C12 myotubes cultured in DM containing 30% 4T1-CM at indicated times (3–48 h). (b) Protein expression of p-Akt^(ser473)^, Akt, p-FoxO3a^(ser253)^, and FoxO3a in C2C12 myotubes cultured for 48 h in DM containing 30% 4T1-CM with or without indicated doses KIC (0.3 mM). Protein expression was evaluated using IB and normalized using Akt and FoxO3a. Each data point represents triplicate measurements (n = 3). (c, d) C2C12 myotubes were pretreated with LY (20 μM) for 1 h, then incubated with DM containing 30% CM with or without KIC (0.3 mM) for 48 h. Relative mRNA expression of *MuRF1*, *MAFbx*, and *myostatin* was analyzed using RT-PCR and normalized to GAPDH. (c) C26-CM treatment. (d) 4T1-CM treatment. Each data point represents triplicate measurements (n = 3). Statistical analysis was performed using one-way ANOVA. *p < 0.05, **p < 0.01, and ***p < 0.001 versus control. ANOVA, analysis of variance; CM, conditioned media; DM, differentiation media; KIC, alpha-ketoisocaproate; LY, LY294002; RT-PCR, real-time polymerase chain reaction; SEM, standard error of the mean.

**Fig. S5.** KIC inhibits myotube atrophy by enhancing p-Akt and FoxO3a interaction and inhibiting FoxO3a translocation in CM-treated C2C12 myotubes. (a, b) C2C12 myotubes were incubated for 48 h in DM containing 30% C26-CM or 4T1-CM, with or without KIC (0.3 mM). Cells were fractionated into nuclear and cytoplasmic fractions, and FoxO3a protein expression was assessed by IB, normalized to GAPDH (cytoplasmic) and Lamin B (nuclear). Each data represents triplicate measurements (n = 3). (c) Localization of FoxO3a was assessed by ICC in C2C12 myotubes incubated in DM containing 30% 4T1-CM, with or without KIC (0.3 mM) for 48 h. Cells were stained for FoxO3a (green) and DAPI (blue) and visualized by confocal microscopy. (d) Protein–protein interactions between 14-3-3 proteins, p-Akt^(ser473)^, and FoxO3a were analyzed in C2C12 myotubes cultured in DM containing 30% 4T1-CM for the indicated times (1–24 h) or DM containing 30% 4T1-CM with or without KIC (0.3 mM) for 24 h. Co-IP with anti-FoxO3a antibody was used to evaluate protein interactions. (e, f) C2C12 myotubes were transfected with Akt or FoxO3a siRNA (100 nM) or control siRNA (100 nM) and incubated for 24 h, followed by treatment with DM containing 30% C26-CM, with or without KIC (0.3 mM) for 48 h. Relative mRNA expression of Akt, FoxO3a, and myostatin was analyzed by RT-PCR and normalized to GAPDH (n = 3). Each data points were expressed as mean ± SEM. Statistical analysis was performed using one-way ANOVA. *p < 0.05, **p < 0.01, and ***p < 0.001 versus control. Scale bar = 50 μm. ANOVA, analysis of variance; CM, conditioned media; Co-IP, co-immunoprecipitation; DM, differentiation media; IB, immunoblot; ICC, immunocytochemistry; KIC, alpha-ketoisocaproate; RT-PCR, real-time polymerase chain reaction; SEM, standard error of the mean.

**Fig. S6.** KIC inhibits myotube atrophy by regulation of myostatin in TCM-treated C2C12 myotubes. (a) Schematic diagram of the preparation of tumor-derived conditioned medium (TCM) from BALB/c derived tumors. C26 or 4T1 cancer cells (1 × 10⁶) were subcutaneously injected into BALB/c mice (step 1), and tumors were allowed to develop for 14 days (step 2). Following tumor formation, tumors were excised (step 3), minced (step 4), enzymatically digested, and filtered (step 5). The resulting single-cell suspension, containing heterogeneous cells from the tumor microenvironment, was cultured (step 6), and the supernatant was collected as TCM (step 7). TCM was applied to C2C12 myotubes from day 5 to day 7 of differentiation to assess the effects of tumor-derived factors on muscle cells. (b-d) C2C12 myotubes were analyzed after incubation for 48 h in DM containing 30% TCM (C26- or 4T1-) with or without KIC (0.3 mM). (b) Comparison of the relative mRNA expression of myostatin was evaluated using RT-PCR and normalized using GAPDH (n = 3). (c) Protein expression of p-Akt^(ser473)^, Akt, p-FoxO3a^(ser253)^, FoxO3a, and myostatin was evaluated using IB and normalized using β-actin (n = 3). (d) MyHC ICC and myotube morphology analysis. MyHC (green) and DAPI (blue) were used for visualization. Myotube diameter was quantified using ImageJ (n = 50). The fusion index was calculated as the percentage of nuclei within MyHC-positive myotubes (n = 5). Data are presented as mean ± SEM. Statistical analysis was performed using one-way ANOVA. *p < 0.05, **p < 0.01, and ***p < 0.001 versus control. Scale bar, 100 μm. ANOVA, analysis of variance; TCM, tumor-derived conditioned media; DM, differentiation media; IB, immunoblot; ICC, immunocytochemistry; KIC, alpha-ketoisocaproate; RT-PCR, real-time polymerase chain reaction; SEM, standard error of the mean; SF, Serum free media.
