# Supplementary material for: Alpha‐Ketoisocaproate Attenuates Muscle Atrophy in Cancer Cachexia Models
Source: J Cachexia Sarcopenia Muscle. 2025 Aug 14;16(4):e70044. doi: 10.1002/jcsm.70044 (PMC12351804; doi:10.1002/jcsm.70044)
Supplement: Supplementary file 3 — Data S3 Supplementary Figures. [file JCSM-16-e70044-s001.pptx]

## Slide 1
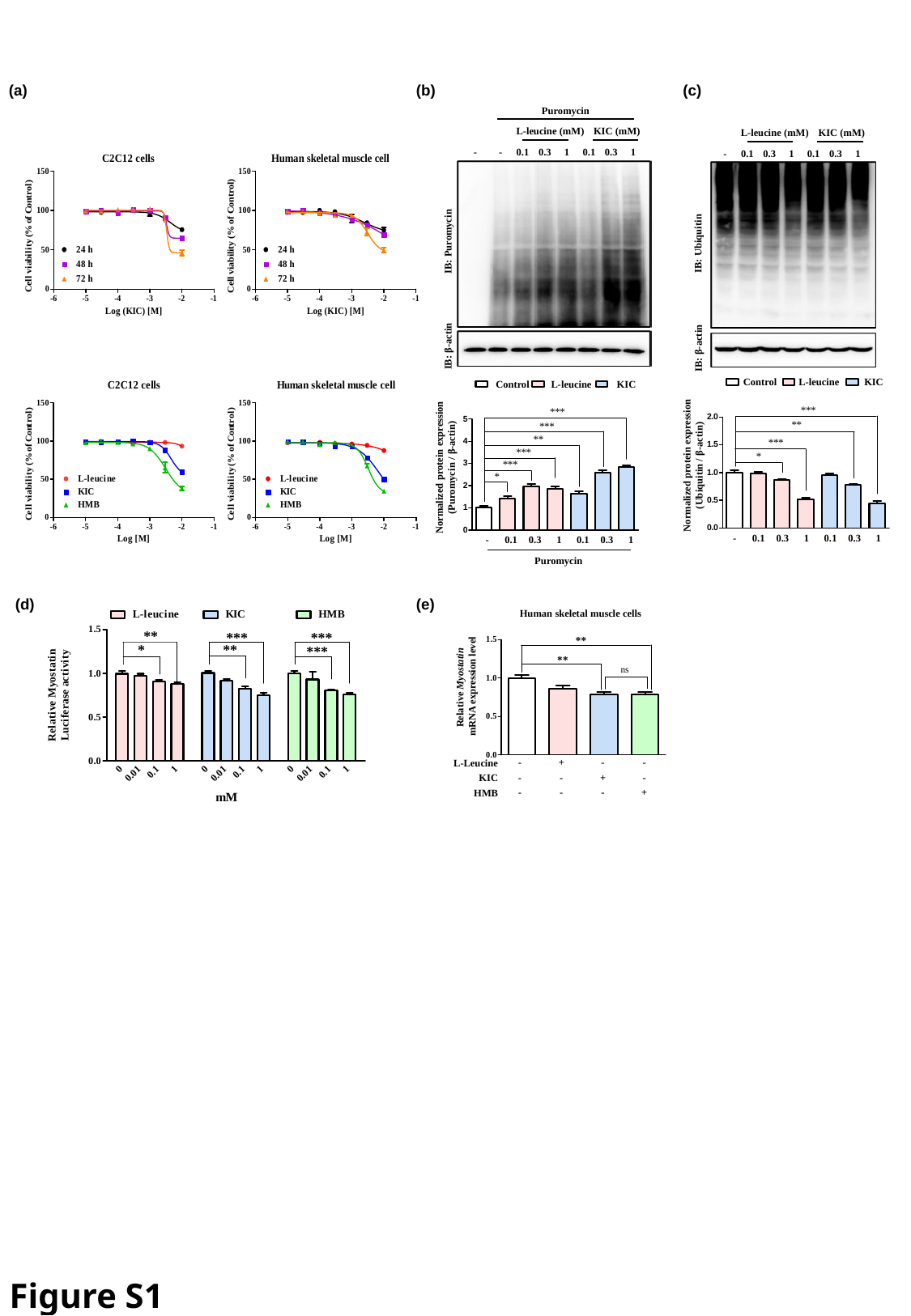

(a)
(b)
(c)
Puromycin
L-leucine (mM)
KIC (mM)
-
0.1
0.3
1
0.1
0.3
1
IB: Puromycin
IB: β-actin
-
L-leucine (mM)
KIC (mM)
-
0.1
0.3
1
0.1
0.3
1
IB: Ubiquitin
IB: β-actin
Control
L-leucine
KIC
Normalized protein expression
(Ubiquitin / β-actin)
-
0.1
0.3
1
0.1
0.3
1
Control
L-leucine
KIC
Normalized protein expression
(Puromycin / β-actin)
-
0.1
0.3
1
0.1
0.3
1
Puromycin
(d)
(e)
Human skeletal muscle cells
-
+
-
-
L-Leucine
-
-
+
-
KIC
-
-
-
+
HMB
Relative Myostatin
mRNA expression level
Figure S1

## Slide 2
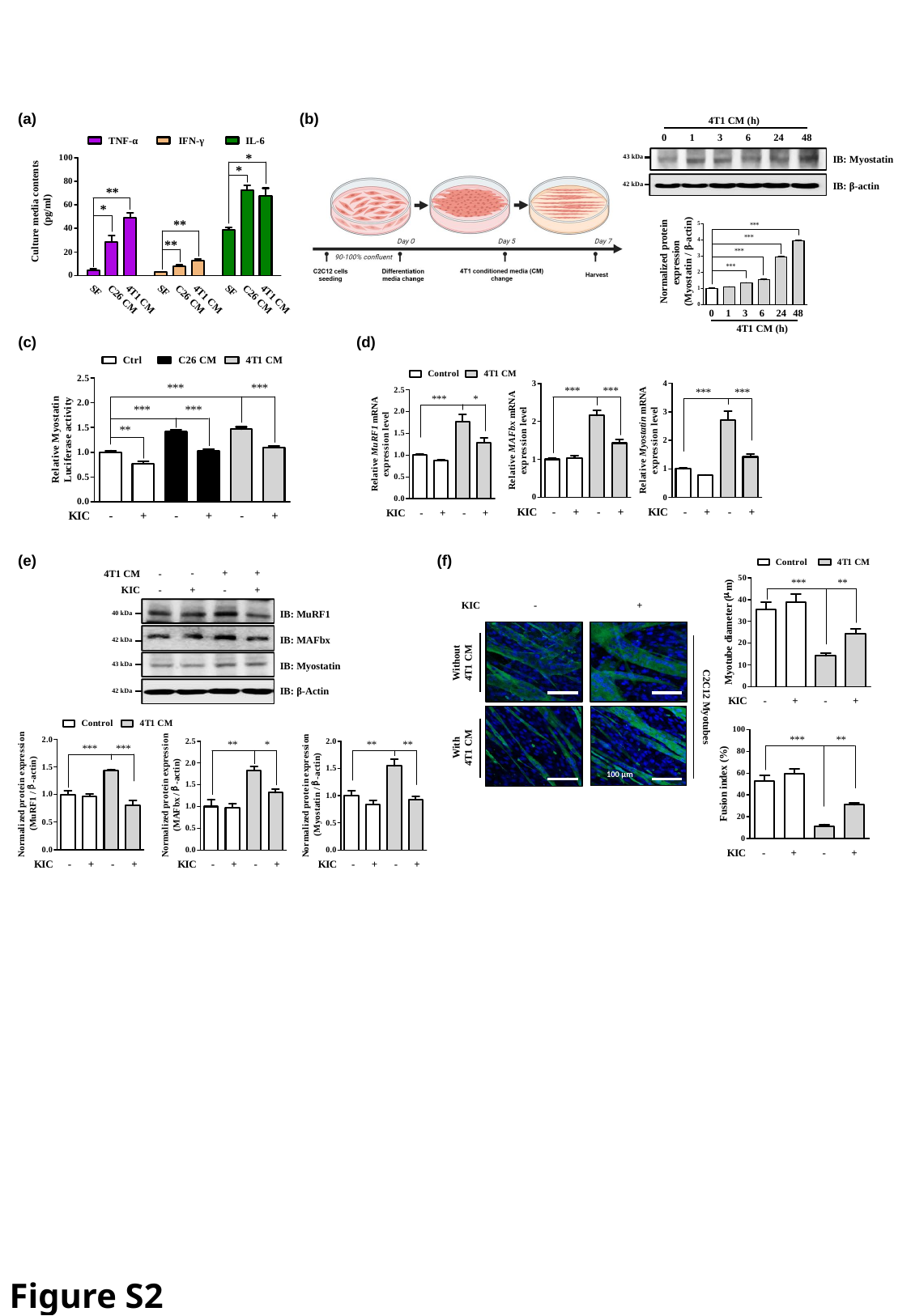

(b)
(a)
4T1 CM (h)
0
1
3
6
24
48
43 kDa
IB: Myostatin
IB: β-actin
42 kDa
Culture media contents
(pg/ml)
TNF-α
IFN-γ
IL-6
SF
C26 CM
4T1 CM
SF
C26 CM
4T1 CM
SF
C26 CM
4T1 CM
Normalized protein
 expression
(Myostatin / β-actin)
0
1
3
6
24
48
4T1 CM (h)
(c)
(d)
(e)
(f)
-
+
+
-
4T1 CM
KIC
-
+
-
+
IB: MuRF1
40 kDa
IB: MAFbx
42 kDa
IB: Myostatin
43 kDa
IB: β-Actin
42 kDa
KIC
-
+
Without 4T1 CM
With 4T1 CM
C2C12 Myotubes
100 μm
Figure S2

## Slide 3
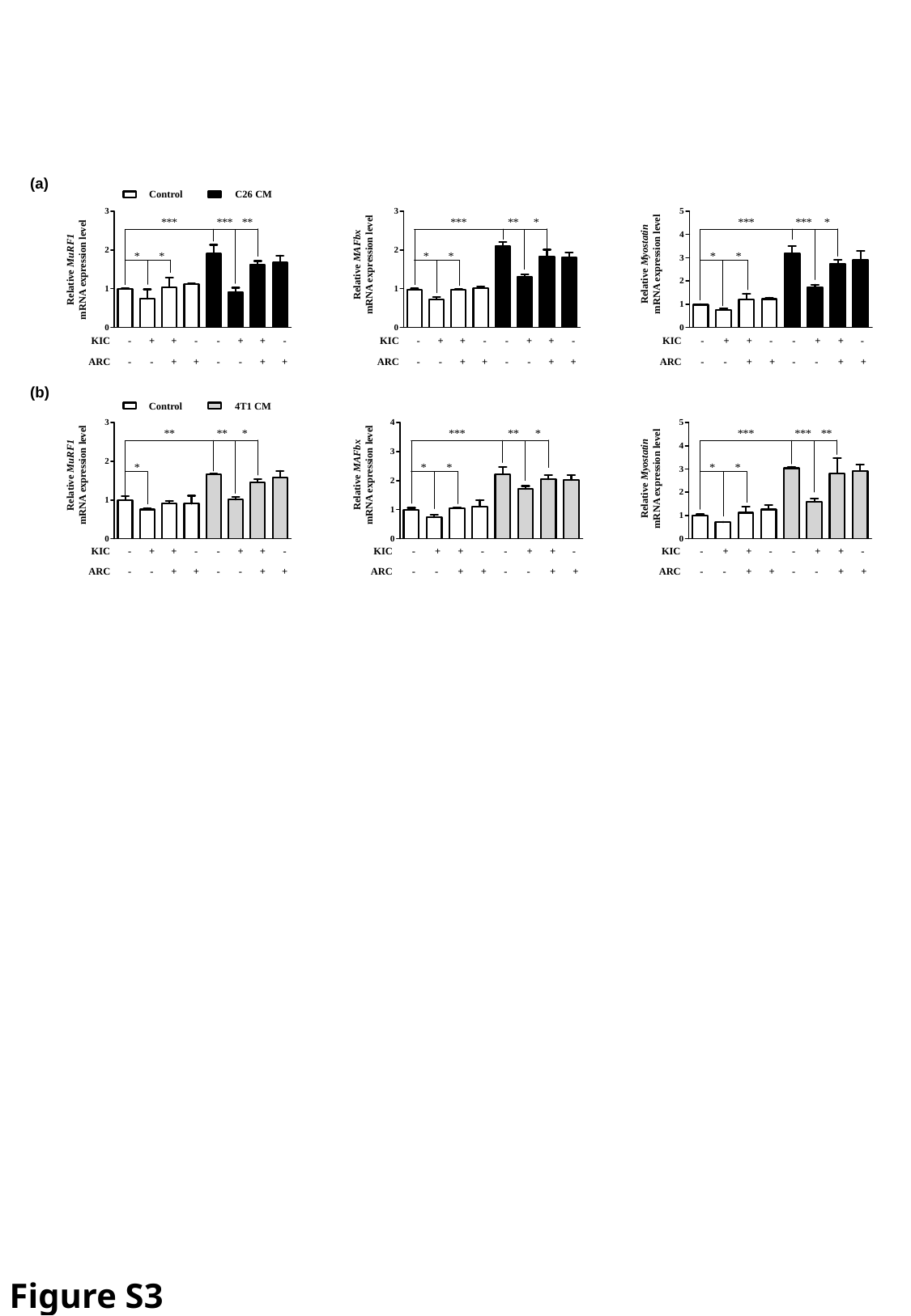

(a)
Control
C26 CM
Relative MuRF1 mRNA expression level
KIC
-
+
+
-
-
+
+
-
ARC
-
-
+
+
-
-
+
+
Relative MAFbx mRNA expression level
KIC
-
+
+
-
-
+
+
-
ARC
-
-
+
+
-
-
+
+
Relative Myostatin mRNA expression level
KIC
-
+
+
-
-
+
+
-
ARC
-
-
+
+
-
-
+
+
(b)
Control
4T1 CM
Relative MuRF1 mRNA expression level
KIC
-
+
+
-
-
+
+
-
ARC
-
-
+
+
-
-
+
+
Relative MAFbx mRNA expression level
KIC
-
+
+
-
-
+
+
-
ARC
-
-
+
+
-
-
+
+
Relative Myostatin mRNA expression level
KIC
-
+
+
-
-
+
+
-
ARC
-
-
+
+
-
-
+
+
Figure S3

## Slide 4
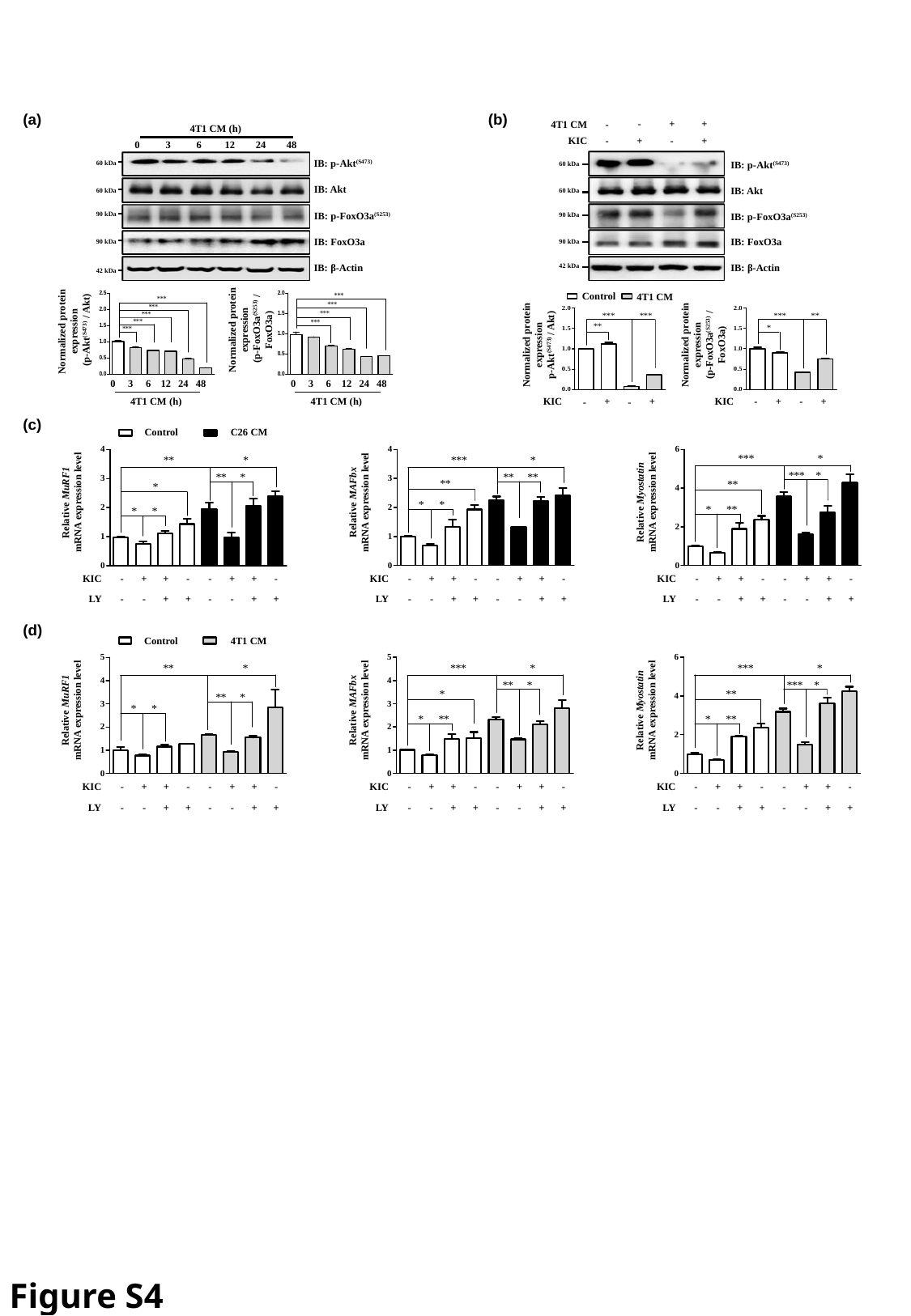

(b)
(a)
-
+
+
-
4T1 CM
KIC
-
+
-
+
IB: p-Akt(S473)
60 kDa
IB: Akt
60 kDa
IB: p-FoxO3a(S253)
90 kDa
IB: FoxO3a
90 kDa
IB: β-Actin
42 kDa
4T1 CM (h)
0
3
6
12
24
48
IB: p-Akt(S473)
IB: Akt
IB: FoxO3a
IB: β-Actin
IB: p-FoxO3a(S253)
60 kDa
60 kDa
90 kDa
90 kDa
42 kDa
Normalized protein
 expression
 (p-FoxO3a(S253) / FoxO3a)
0
3
6
12
24
48
4T1 CM (h)
Normalized protein
 expression
 (p-Akt(S473) / Akt)
0
3
6
12
24
48
4T1 CM (h)
Control
4T1 CM
Normalized protein
 expression
p-Akt(S473) / Akt)
KIC
+
+
-
-
Normalized protein
 expression
(p-FoxO3a(S253) / FoxO3a)
KIC
-
+
-
+
(c)
Control
C26 CM
Relative MuRF1 mRNA expression level
KIC
-
+
+
-
-
+
+
-
LY
-
-
+
+
-
-
+
+
Relative MAFbx mRNA expression level
KIC
-
+
+
-
-
+
+
-
LY
-
-
+
+
-
-
+
+
Relative Myostatin mRNA expression level
KIC
-
+
+
-
-
+
+
-
LY
-
-
+
+
-
-
+
+
(d)
Control
4T1 CM
Relative MuRF1 mRNA expression level
KIC
-
+
+
-
-
+
+
-
LY
-
-
+
+
-
-
+
+
Relative MAFbx mRNA expression level
KIC
-
+
+
-
-
+
+
-
LY
-
-
+
+
-
-
+
+
Relative Myostatin mRNA expression level
KIC
-
+
+
-
-
+
+
-
LY
-
-
+
+
-
-
+
+
Figure S4

## Slide 5
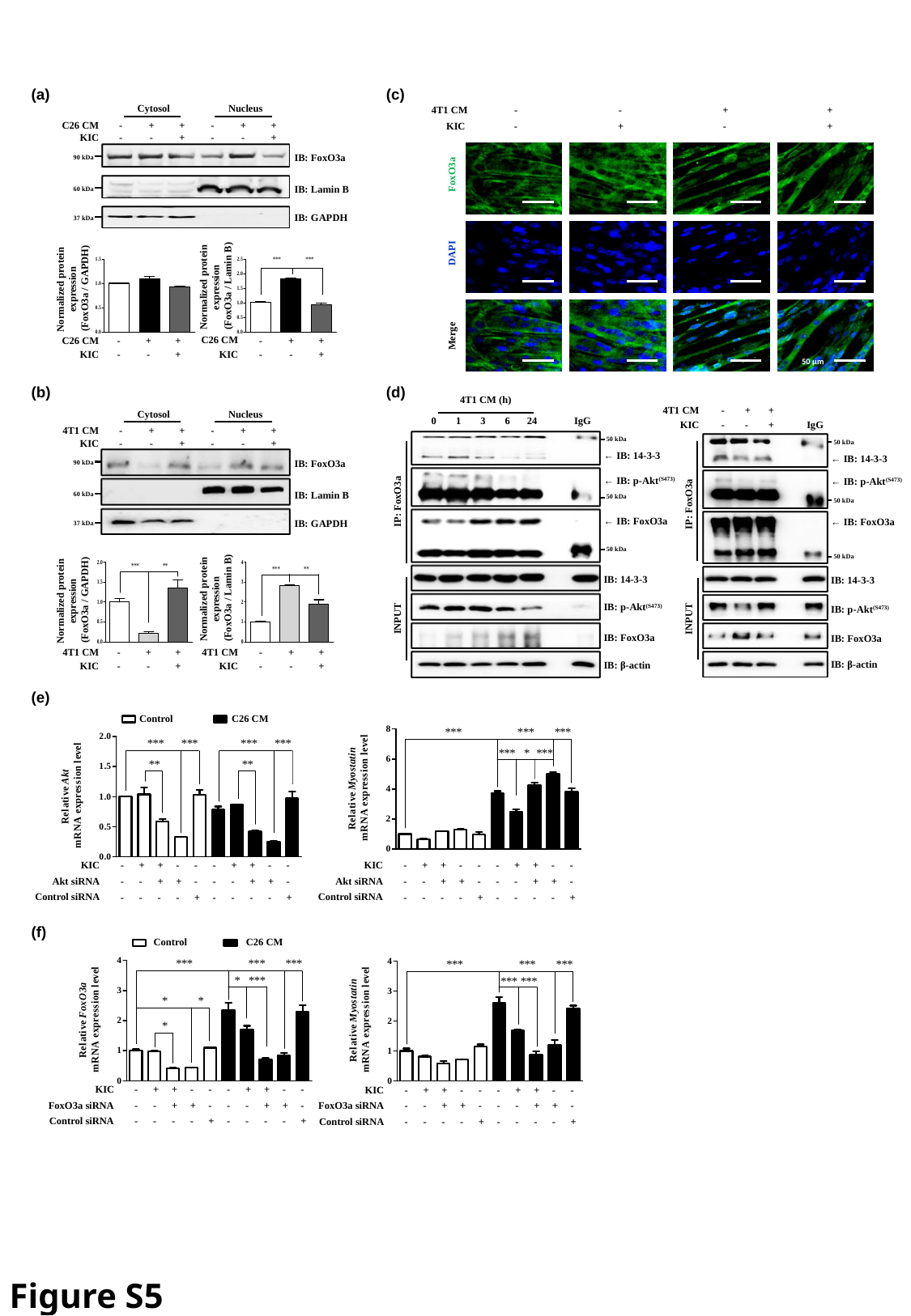

(c)
(a)
Cytosol
Nucleus
C26 CM
-
+
+
-
+
+
KIC
-
-
+
-
-
+
IB: FoxO3a
90 kDa
IB: Lamin B
60 kDa
IB: GAPDH
37 kDa
4T1 CM
-
-
+
+
KIC
-
+
-
+
FoxO3a
DAPI
Merge
50 μm
Normalized protein
 expression
 (FoxO3a / Lamin B)
C26 CM
-
+
+
KIC
-
-
+
Normalized protein
 expression
 (FoxO3a / GAPDH)
C26 CM
-
+
+
-
-
+
KIC
(b)
(d)
4T1 CM (h)
0
1
3
6
24
IgG
50 kDa
IP: FoxO3a
← IB: 14-3-3
← IB: p-Akt(S473)
50 kDa
← IB: FoxO3a
50 kDa
IB: 14-3-3
INPUT
IB: p-Akt(S473)
IB: FoxO3a
IB: β-actin
4T1 CM
-
+
+
KIC
-
-
+
IgG
50 kDa
← IB: 14-3-3
← IB: p-Akt(S473)
50 kDa
IP: FoxO3a
← IB: FoxO3a
50 kDa
IB: 14-3-3
IB: p-Akt(S473)
INPUT
IB: FoxO3a
IB: β-actin
Cytosol
Nucleus
4T1 CM
-
+
+
-
+
+
KIC
-
-
+
-
-
+
IB: FoxO3a
90 kDa
IB: Lamin B
60 kDa
IB: GAPDH
37 kDa
Normalized protein
 expression
 (FoxO3a / Lamin B)
4T1 CM
-
+
+
KIC
-
-
+
Normalized protein
 expression
 (FoxO3a / GAPDH)
4T1 CM
-
+
+
-
-
+
KIC
(e)
Control
C26 CM
-
-
+
+
-
-
-
-
+
+
-
-
+
+
+
+
-
-
-
-
+
+
-
-
-
-
-
-
-
-
KIC
Akt siRNA
Control siRNA
-
-
+
+
-
-
-
-
+
+
-
-
+
+
+
+
-
-
-
-
+
+
-
-
-
-
-
-
-
-
KIC
Akt siRNA
Control siRNA
(f)
Control
C26 CM
-
-
+
+
-
-
-
-
+
+
-
-
+
+
+
+
-
-
-
-
+
+
-
-
-
-
-
-
-
-
KIC
FoxO3a siRNA
Control siRNA
-
-
+
+
-
-
-
-
+
+
-
-
+
+
+
+
-
-
-
-
+
+
-
-
-
-
-
-
-
-
KIC
FoxO3a siRNA
Control siRNA
Figure S5

## Slide 6
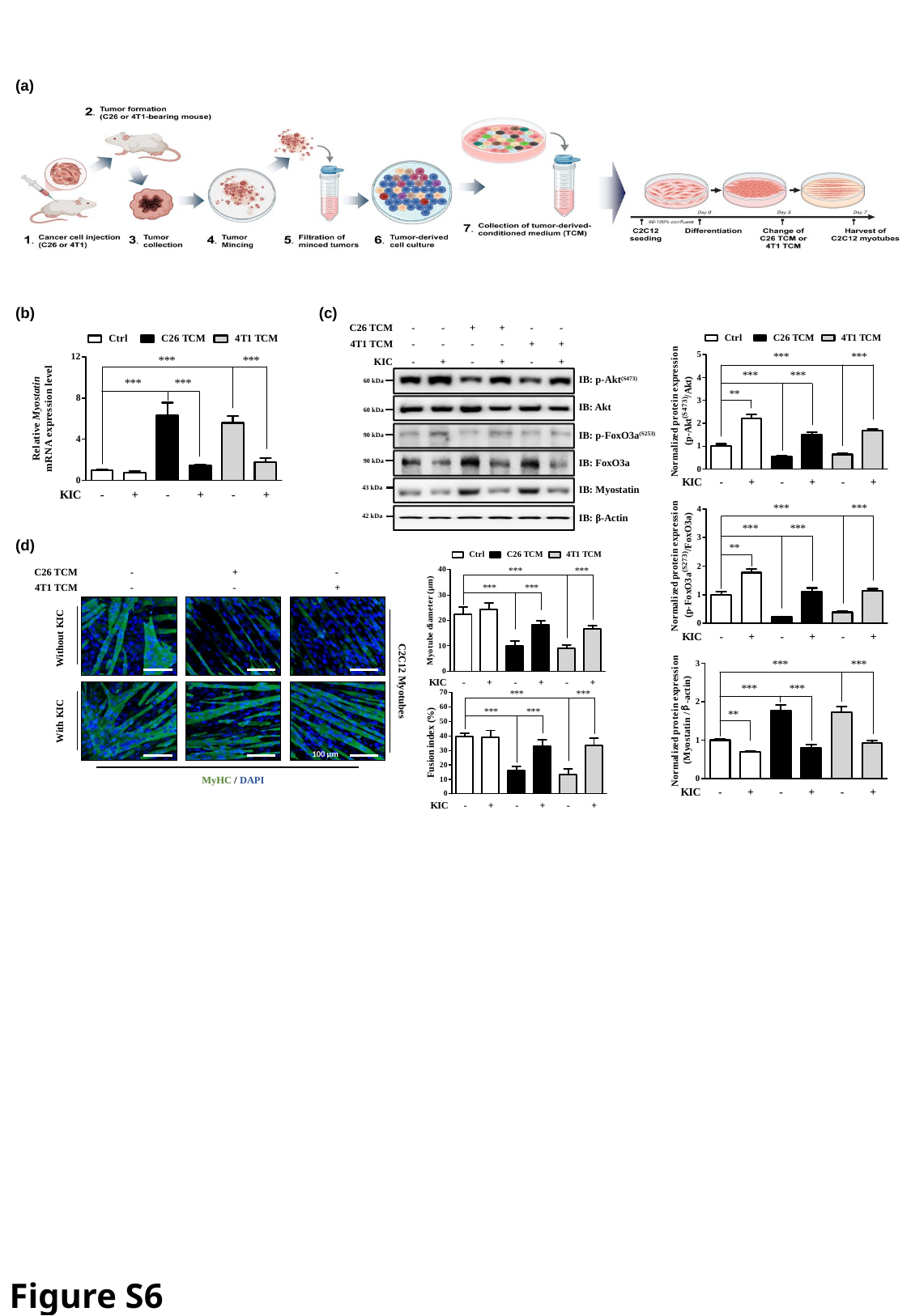

(a)
(c)
(b)
C26 TCM
-
-
+
+
-
-
4T1 TCM
-
-
-
-
+
+
KIC
-
+
-
+
-
+
IB: p-Akt(S473)
60 kDa
IB: Akt
60 kDa
IB: p-FoxO3a(S253)
90 kDa
IB: FoxO3a
90 kDa
IB: Myostatin
43 kDa
IB: β-Actin
42 kDa
(d)
-
+
-
C26 TCM
4T1 TCM
-
-
+
Without KIC
With KIC
C2C12 Myotubes
100 μm
MyHC / DAPI
Figure S6
