# Supplementary material for: Alpha‐Ketoisocaproate Attenuates Muscle Atrophy in Cancer Cachexia Models
Source: J Cachexia Sarcopenia Muscle. 2025 Aug 14;16(4):e70044. doi: 10.1002/jcsm.70044 (PMC12351804; doi:10.1002/jcsm.70044)
Supplement: Supplementary file 4 — Table S1 Nucleotide sequences of primers and the operating condition of RT‐PCR. [file JCSM-16-e70044-s003.docx]

**Table S1.** Nucleotide sequences of primers and the operating condition of RT-PCR

| **Primer** | | **Nucleotide sequence (5 -> 3)** | **AT** |  |
| --- | --- | --- | --- | --- |
| ***GAPDH*** (human) | forward | 5'-GTG AAG GTC GGA GTC AAC G-3′ | 58℃ |  |
|  | reverse | 5'-TTG ACG GTG CCA TGG AAT TT-3′ |  |  |
| ***Myostatin*** (human) | forward | 5′-TGA GAA TGG TCA TGA TCT TGC TGT-3′ | 58℃ |  |
|  | reverse | 5′-TCA TCA CAG TCA AGA CCA AAA TCC-3′ |  |  |
| ***MuRF1*** (human) | forward | 5′-GAA TGA GAG GCC CCC AGA TG-3′ | 58℃ |  |
|  | reverse | 5′-ACC CTA GTC CCT GCT CTC TG-3′ |  |  |
| ***MAFbx*** (human) | forward | 5′-CTG ACC TGC CTT TGT GCC TA-3′ | 58℃ |  |
|  | reverse | 5′-CTC GGA GAA GTG GTA CTG GC-3′ |  |  |
| ***GAPDH*** (mouse) | forward | 5′-GTG TTC CTA CCC CCA ATG TG-3′ | 58℃ |  |
|  | reverse | 5′-CCT GCT TCA CCA CCT TCT TG-3′ |  |  |
| ***Myostatin*** (mouse) | forward | 5′-GCA CTG GTA TTT GGC AGA GT-3′ | 58℃ |  |
|  | reverse | 5′-TTC AGC CCA TCT TCT CCT GG-3′ |  |  |
| ***MuRF1*** (mouse) | forward | 5′-GTC CAT GTC TGG AGG TCG TT-3′ | 58℃ |  |
|  | reverse | 5′-AGG AGC AAG TAG GCA CCT CA-3′ |  |  |
| ***MAFbx*** (mouse) | forward | 5′-ATG CAC ACT GGT GCA AAG AG-3′ | 58℃ |  |
|  | reverse | 5′-TGT AAG CAC ACA GGC AGG TC-3′ |  |  |
| ***Akt*** (mouse) | | forward | 5′-AAT GTG GGC TCA TGG GTC TG-3′ | 58℃ |
|  |  | reverse | 5′-AGA GGG AGA GGG CCA GTT AG-3′ |  |
| ***FoxO3a*** (mouse) | forward | 5′-AGC CGT GTA CTG TGG AGC TT-3′ | 58℃ |  |
|  | reverse | 5′-TCT TGG CGG TAT ATG GGA AG-3′ |  |  |
| ***MCT1*** (mouse) | forward | 5′-CAG TGT TAG TCG GAG CCT TC-3′ | 58℃ |  |
|  | reverse | 5′-TAC TTC ACT GGT CGT TGC AC-3′ |  |  |
| ***MCT2*** (mouse) | forward | 5′-CAG CTT TGG TGG TCT ACG TT-3′ | 58℃ |  |
|  | reverse | 5′-ACT GGA CAA CAC TCC ACG AT-3′ |  |  |

**T**: thymine, **A**: adenine, **C**: cytosine, **G**: guanine, **AT**: Annealing temperature
